# Effects of Self-Guided Digital Mindfulness Interventions on the Mental Health of Health Care Workers: Systematic Review and Meta-Analysis of Randomized Controlled Trials

**DOI:** 10.2196/74272

**Published:** 2026-08-21

**Authors:** Natsu Sasaki, Akiomi Inoue, Mako Iida, Kazuhiro Watanabe, Yuka Kobayashi, Asuka Sakuraya, Hisashi Eguchi, Kanami Tsuno, Yu Komase, Yasumasa Otsuka, Mai Iwanaga, Reiko Inoue, Kazuto Kuribayashi, Ayako Hino, Hiroki Asaoka, Satoru Kanamori, Yuta Inagawa, Takenori Yamauchi, Hiroki Ikeda, Go Muto, Rika Kato, Takuro Okuyama, Naomichi Tani, Noriko Kojimahara, Takeshi Ebara, Akihito Shimazu, Akizumi Tsutsumi, Norito Kawakami, Kotaro Imamura

**Affiliations:** 1 Department of Mental Health, Graduate School of Medicine, The University of Tokyo, Tokyo, Japan; 2 Institutional Research Center, University of Occupational and Environmental Health, Japan, Kitakyushu, Japan; 3 Department of Public Health, Kitasato University School of Medicine, Sagamihara, Japan; 4 Department of Clinical Psychology, Faculty of Social Policy and Administration, Hosei University, Tokyo, Japan; 5 Department of Digital Mental Health, Graduate School of Medicine, The University of Tokyo, 7-3-1 Hongo, Bunkyo-ku, Tokyo, 113-0033, Japan, 81 3-5841-3521; 6 Department of Mental Health, Institute of Industrial Ecological Sciences, University of Occupational and Environmental Health, Japan, Kitakyushu, Japan; 7 School of Health Innovation, Kanagawa University of Human Services, Kawasaki, Japan; 8 Healthcare Business Division, Fujitsu Japan Limited, Kawasaki, Japan; 9 Institute of Human Sciences, University of Tsukuba, Tokyo, Japan; 10 Department of Community Mental Health and Law, National Institute of Mental Health, National Center of Neurology and Psychiatry, Tokyo, Japan; 11 Department of Psychiatric Nursing, Division of Nursing, Wayo Women’s University, Ichikawa, Japan; 12 Graduate School of Public Health, Teikyo University, Tokyo, Japan; 13 Department of Psychiatry, Jichi Medical University, Shimotsuke, Japan; 14 Department of Hygiene, Public Health and Preventive Medicine, Showa Medical University, Tokyo, Japan; 15 National Institute of Occupational Safety and Health, Japan Organization of Occupational Health and Safety, Kawasaki, Japan; 16 Department of Hygiene and Public Health, Tokyo Women's Medical University School of Medicine, Tokyo, Japan; 17 Department of Ergonomics, Institute of Industrial Ecological Sciences, University of Occupational and Environmental Health, Japan, Kitakyushu, Japan; 18 Section of Epidemiology, Shizuoka Graduate University of Public Health, Shizuoka, Japan; 19 Faculty of Policy Management, Keio University, Kanagawa, Japan

**Keywords:** digital intervention, occupational stress, psychological intervention, self-guided intervention, telehealth

## Abstract

**Background:**

Mindfulness interventions are considered an effective strategy for preventing mental health problems among health care workers (HCWs). While prior reviews have often included facilitator-guided or multicomponent interventions, it is unclear whether self-guided digital mindfulness interventions can also be effective without professional or in-person support.

**Objective:**

This systematic review aimed to investigate the effects of self-guided digital mindfulness interventions on the mental health and well-being of HCWs.

**Methods:**

This work was conducted as part of the DeLiGHT project. We searched electronic databases, including PubMed (MEDLINE), Embase, Cochrane CENTRAL, PsycINFO, PsycARTICLES, and the Japan Medical Abstract Society database, from inception in 2010 to July 25, 2023. To ensure timeliness, we performed an updated search on December 27, 2025. Database searching yielded 37,851 abstracts and 145 studies that examined the effectiveness of digital health interventions for workers in the initial 2023 screen. In the present study, we limited the included studies to those that met the following criteria: randomized controlled trials (RCTs) using self-guided digital mindfulness interventions (eg, smartphone apps or web-based programs) delivered without face-to-face or professional support compared to waiting list controls or usual care among HCWs. The updated search in 2025 yielded 6328 additional records, of which 11 studies were examined. Study quality was assessed using the Cochrane Risk of Bias tool, with meta-analyses conducted using a random-effects model.

**Results:**

A total of 9 RCTs with 3088 participants were included in the final analysis. The study populations were diverse, covering North America, Europe, Oceania, and Asia. Interventions were delivered via various digital platforms, including commercial mindfulness apps (n=3), social networking service tools (n=3), and web-based programs (n=3), with an average duration of 6.17 weeks (range: 1.5‐18 wk). Five studies assessed outcomes only postintervention, and 4 included short-term follow-up (4‐12 wk); no long-term effects were evaluated. The meta-analysis showed a significant beneficial effect on depression (standardized mean difference [SMD]=−0.44, 95% CI −0.88 to −0.00; *P*=.049), anxiety (SMD=−0.29, 95% CI −0.51 to −0.06; *P*=.011), perceived stress (SMD=−0.42, 95% CI −0.77 to −0.06; *P*=.02), and well-being (SMD=0.20, 95% CI 0.09 to 0.30; *P*<.001) immediately postintervention. The adherence rates reported across studies were highly variable, ranging from 19% to 56%. According to the Cochrane Risk of Bias tool, most studies were rated as having some concerns or a high risk of bias. The certainty of evidence included in the analysis ranged from “Low” to “Very Low.”

**Conclusions:**

Overall, low to very low certainty evidence suggests that self-guided digital mindfulness interventions may have short-term beneficial effects on depression, anxiety, perceived stress, and well-being among HCWs. Future high-quality studies are needed to examine the long-term effects and cross-cultural generalizability of these interventions.

## Introduction

Health care workers (HCWs) are at high risk of experiencing mental health problems worldwide [1,2]. A systematic review has reported that the prevalence of mental health symptoms is high among HCWs: depression (13%), anxiety (16%), and traumatic stress (22%) [3]. Another systematic review reported that half of HCWs experience burnout [4], characterized primarily by emotional exhaustion and compassion fatigue, which negatively impacts patient care [5]. These mental health challenges affect not only the well-being of HCWs but also the quality of care provided to patients [6], potentially leading to long-term negative consequences on community health care systems. Therefore, effective and accessible interventions that focus on individual-level approaches, which are likely easier to implement, are urgently needed to improve the mental health of HCWs.

Mindfulness interventions are an effective nonpharmacological approach for improving mental health [7]. Mindfulness refers to paying attention to present-moment experiences in a nonjudgmental and accepting manner [8]. In particular, there is a growing interest in self-guided digital mindfulness interventions, which do not require face-to-face delivery or professional support, as scalable and accessible approaches. Systematic reviews have shown the effectiveness of face-to-face mindfulness interventions in improving the mental health of HCWs, including individuals with depression, anxiety [9,10], and emotional exhaustion [11], as well as in promoting their well-being [12]. Mindfulness is the most commonly used psychological group intervention for reducing distress symptoms in HCWs [13]. Mindfulness is an acceptable intervention for HCWs because it has a reasonable rationale and can be done quickly [14]. However, HCWs often face barriers when engaging in mental health interventions, such as limited availability (eg, shift work), high job demands, concerns about stigma, and negative social judgments, as well as issues related to disclosure and confidentiality, which are more common among HCWs than other workers [15]. In this context, self-guided digital interventions, which are not provided by professionals or delivered in person, may have advantages in engaging HCWs in the interventions.

Digital mindfulness interventions can overcome the barriers of logistics and time availability by providing flexible access and standardized content at a low cost [16]. Self-guided digital mindfulness tools have already been widely used, and over 560 mindfulness apps are available on the market [17]. However, the effectiveness of self-guided digital mindfulness interventions on the mental health of HCWs is still unclear. A previous systematic review reported on the effectiveness of web-based mindfulness-based interventions for HCWs. However, it focused primarily on the COVID-19 pandemic, with half of the included studies conducted in a single country, leading to a risk of overestimation and limited generalizability [18].

It did not restrict itself to self-guided interventions and included nonmindfulness components such as cognitive behavioral therapy, music therapy, and positive psychology interventions. Additionally, another review included studies involving students, searched only a single database, and did not conduct any quantitative analyses of the effects of web-based tools and mobile apps [19]. Considering advancements in technology and synthesizing the findings of randomized controlled trials (RCTs) conducted over the past 10 years with participants limited to HCWs, and not restricted to the pandemic period, may provide a more comprehensive and accurate understanding of the effectiveness of digital mindfulness interventions on the mental health of HCWs. If self-guided digital mindfulness interventions are found to be effective, such interventions may be promoted even in emergency situations like pandemics and in low-resource settings.

This systematic review and meta-analysis thus aimed to address this gap by examining the effectiveness of self-guided digital mindfulness interventions without face-to-face, professional, or personal support components on mental health and well-being among HCWs from 2010 to 2025.

## Methods

### Study Design

This work was part of an overarching large-scale systematic review project developing Minds-compliant guidelines for general preventive interventions using digital health technologies for mental health (the DeLiGHT project; [20]), funded by the Japan Agency for Medical Research and Development.

The review protocol has been registered in the UMIN registry (registration number: UMIN000051631). The method was reported according to PRISMA (the Preferred Reporting Items for Systematic Review and Meta-Analysis) 2020 guidelines (Checklist 1) [21]. Among the included literature in the DeLiGHT project, this review reported on the effectiveness of fully automated digital mindfulness interventions for HCWs.

### Data Sources, Search Strategy, and Study Selection Process

In the DeLiGHT project, we initially searched for publications from 2010 to July 25, 2023, across databases such as PubMed (MEDLINE), Embase, Cochrane CENTRAL, PsycINFO, PsycARTICLES, and the Japan Medical Abstracts Society database. The search was limited to studies published from 2010 onward, reflecting the widespread adoption of smartphones and web-based platforms. The search terms are detailed in Multimedia Appendix 1. Search terms were comprehensively selected, referring to previous reports on digital health technology [22] and outcomes [23]. The search strategy was constructed using combinations of population- and intervention-related terms. In addition, study design-related words were applied to restrict the search to randomized controlled trials. Although outcome-related terms were included to improve feasibility given the broad scope of the literature, we acknowledge that the inclusion of outcome terms may increase the risk of missing relevant studies, in line with the recommendations of the Cochrane Handbook [24,25]. We included all studies that followed the inclusion criteria of participants, interventions, comparisons, and outcomes (PICO): (P) any type of employee or worker; (I) any type of nonpharmacological intervention aiming at primary prevention using digital health technologies (eg, exercises, diet, lifestyle, and psychological interventions such as cognitive behavioral therapy); (C) any type of control condition; and (O) mental health conditions, positive mental health, and work-related outcomes. In the subgroup considerations, HCWs were defined using terms explicitly referring to medical and health professionals (eg, physicians, nurses, pharmacists, psychologists, therapists, and health care providers). In addition, studies using an RCT design and published after 2010 were included. The language was limited to English or Japanese. Exclusion criteria were as follows: (1) studies targeting participants who have any specific disorder, (2) studies including participants who are not employees or workers, (3) studies applying any interventions aimed at secondary or tertiary prevention, and (4) studies aiming to evaluate the treatment effects of interventions on specific disorders or symptoms. One investigator (M Iida) removed duplicate entries, and the remaining articles were shared with all co-authors. During the first screening phase, based on the eligibility criteria, each article’s title and abstract were independently assessed by pairs of investigators, with one investigator being a staff member from an external contracting agency. Following this, 2 investigators independently reviewed the full texts of studies that passed the first screening. Any disagreements were resolved through discussion and consensus among all authors. Reasons for study exclusion were documented during the full-text review phase. Microsoft Excel was used to manage all identified studies.

The DeLiGHT project’s database search initially retrieved 37,851 abstracts (from 2010 to July 25, 2023). During the initial screening, after removing 15,485 duplicates and 3275 older studies published before 2010, 19,091 records remained for further review, of which 18,806 were excluded. Subsequently, 285 records were forwarded for full-text screening. Following the full-text review, 140 studies were excluded. Finally, 145 RCTs were identified as digital interventions aimed at improving workers’ mental health.

An updated search was conducted on December 27, 2025, in PubMed (MEDLINE), Cochrane CENTRAL, PsycINFO, PsycARTICLES, and the Japan Medical Abstracts Society database, using the same search terms (Multimedia Appendix 1). Notably, Embase could not be used for the updated search due to access restrictions. However, even in the original search, which included Embase, all studies ultimately included in this review were indexed in PubMed. Moreover, previous research has suggested that the additional benefit of supplementing PubMed with Embase may be limited in some fields [26]. Taken together, these considerations suggest that the absence of Embase is unlikely to have materially affected the comprehensiveness of the update. The search was limited to studies published from 2023 onward, to update the search of the DeLiGHT project. In the 2025 update, eligible RCTs were directly identified in accordance with the selection criteria of this study, which are described in the next section.

### Selection Criteria for the Review of Digital Mindfulness Interventions

In this study, we limited the included studies according to the criteria for this review: (P) HCWs, including workers in clinical hospitals or emergency units; (I) digital mindfulness interventions; and (O) mental health symptoms (ie, depression, anxiety, perceived stress, traumatic stress reaction, and sleep quality), emotional exhaustion, compassion fatigue, and positive mental health and well-being. Digital mindfulness interventions in this review were defined as technology-assisted mindfulness interventions aimed at fostering greater attention to and awareness of present-moment experiences [7,27], delivered via digital platforms without face-to-face components or professional or personal support, as described in previous reviews of digital mindfulness interventions [28,29]. Following this definition, we excluded interventions primarily focused on other approaches (eg, cognitive behavioral therapy-based skills) or interventions that could not be evaluated for the extent to which they emphasized mindfulness. Complex or multimodal interventions were carefully discussed in terms of eligibility by NS, AI, and KI. For multimodal interventions, eligibility was determined based on whether mindfulness was described as the primary intervention component in the intervention rationale and content descriptions, with decisions made through independent review and consensus among the reviewers. The yoga component was included in this review if it was delivered via a digital tool without professional support, as yoga has been recognized as a primary discipline and practice with the potential to cultivate mindfulness [30].

### Data Collection Process and Data Items

Among the 145 records identified in the initial screening (DeLiGHT project, from 2010 to July 25, 2023), 3 investigators (NS, AI, and KI) assessed the studies’ eligibility in pairs. In the update, 2 investigators (NS and KI) conducted both the initial and full-text reviews in accordance with the current studies’ eligibility criteria (ie, mindfulness for HCWs). They consolidated the data, which included information such as publication year, authors’ names, study location, participants’ characteristics (eg, HCWs), number of participants, intervention details, control group information, outcome variables, and adherence or dose. Follow-up assessments were defined as outcome measurements conducted after the immediate postintervention assessment, and were used to evaluate the short-term sustainability of intervention effects on the same mental health outcomes.

### Risk of Bias Assessment

Six investigators assessed the risk of bias independently in 3 pairs (NS, AI, M Iida, KW, YKob, and KI) using the recently revised Cochrane risk-of-bias tool for randomized trials (RoB 2.0) [31]. This tool comprises 5 bias domains: bias arising from the randomization process (domain 1), bias due to deviations from intended interventions (domain 2), bias due to missing outcome data (domain 3), bias in measuring the outcome (domain 4), and bias in the selection of the reported result (domain 5). For each domain, a series of signaling questions with answers (yes, probably yes, no information, probably no, and no) determine the risk of bias (low risk, some concerns, and high risk). Any discrepancies in the quality assessment were addressed through discussion and consensus among the authors.

### Synthesis of Results and Meta-Analysis

In the analysis, we synthesized all of the included interventions and outcomes. A meta-analysis was performed if at least 3 eligible studies had the same outcome. The effect size was calculated based on the difference in the change of baseline scores between the intervention and control. The standardized mean difference (SMD) was used to assess the effect of interventions. Meta-analyses were performed for the mean effect on each outcome of mental health symptoms (eg, depression, anxiety, and perceived stress), emotional exhaustion and compassion fatigue, and each outcome of positive mental health and well-being. Meta-analysis was performed at each time point (immediately postintervention and follow-up) for each outcome. A random-effects model was used to consider differences in the treatment effect from study to study. When the SD of change from baseline was not shown, it was calculated based on the assumption that the correlation between scores at baseline and those at follow-up was 0.5 [32]. Heterogeneity between studies was measured through the *I*^2^ statistic (low, moderate, and high heterogeneity corresponding to *I*^2^ values of 25%, 50%, and 75%, respectively) [33]. Funnel plots and Egger tests were used to assess potential publication bias for the meta-analyses [34,35]. Consistent with the Cochrane Handbook [36], tests for funnel plot asymmetry were interpreted with caution because fewer than 10 studies were included, and statistical power is known to be low under such conditions. For outcomes with *I*^2^ more than 75%, we performed sensitivity analyses by excluding outliers identified through visual inspection of funnel plots.

We used the Grading of Recommendations Assessment, Development, and Evaluation (GRADE) approach to assess the certainty of the evidence [37].

Statistical significance was set at a 2-sided *P*<.05. Analysis was conducted using IBM SPSS Statistics Version 28.0. (Japanese version). Meta-analysis was performed in R version 4.5.1 (R Foundation for Statistical Computing) with the *metafor* package [38].

## Results

### Database Searching

**Figure 1.**
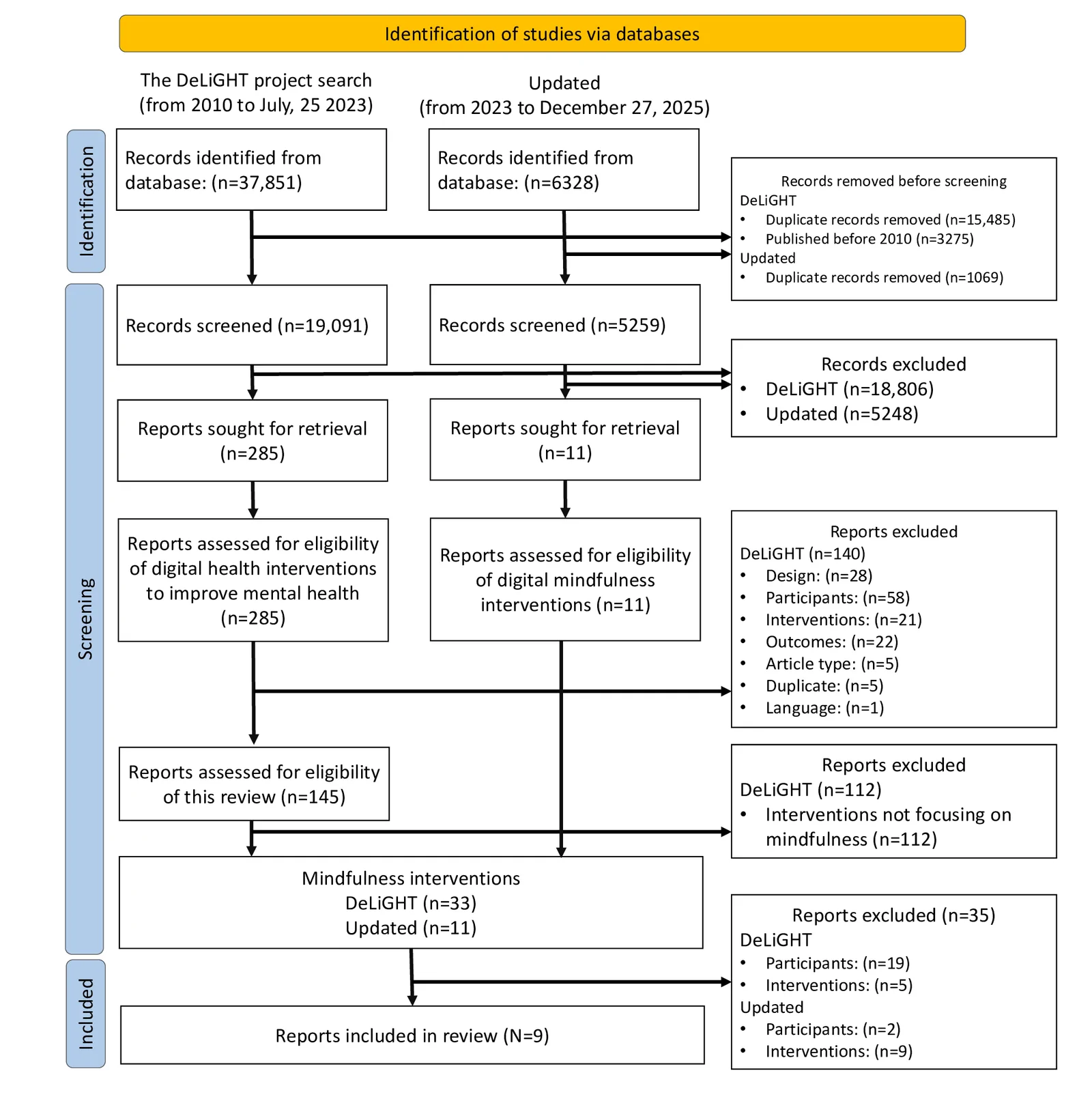
Preferred Reporting Items for Systematic Reviews and Meta-Analyses (PRISMA) 2020 flow diagram of the systematic review search results. DeLiGHT: Developing minds-compliant guidelines for general preventive intervention using digital health technologies for mental health.

Among the 145 studies identified in the DeLiGHT project, we excluded 112 articles because their interventions did not focus on mindfulness. Among 33 mindfulness intervention studies for workers, we excluded 24 studies for reasons such as participants (ie, not targeting HCWs; n=19) and interventions (ie, digital mindfulness without personal or in-person support; n=5). An updated database search retrieved 6328 abstracts (from 2023 to December 27, 2025), and 5259 abstracts were screened after removing duplicate records. Subsequently, 11 studies were assessed for eligibility for digital mindfulness interventions, but all studies were excluded. Finally, 9 studies [39-47] were included in the systematic review. The study selection process is illustrated in Figure 1. A list of 35 studies excluded at full-text screening, along with reasons for exclusion, is provided in Multimedia Appendix 2.

### Study Characteristics

The characteristics of 9 RCTs are presented in Table 1. The total number of included participants was 3088 (ranging from 41 to 2182 in 1 study) [39-47]. Countries where studies were conducted varied, including Western countries (the United States or Canada [42,45]), England [46], Australia [47], Iran [39,44], and other Asian countries (South Korea [40], Singapore [41], and Thailand [43]). Of the 9 included studies, 3 studies [40,42,46] delivered the intervention before the COVID-19 pandemic.

**Table 1.** The details of the included randomized controlled trials (N=9).^a^

First author, year	Intervention year	Country	Participants (total)	Intervention (1-7)^b^	Control	Post FU^c^ (from postsurvey)	Outcomes	Adherence or dose
Asadollah et al [39], 2023	No information but under COVID-19 pandemic	Iran	NICU^d^ nurses in 2 educational hospitals (N=66)	6 weeks Reduction of compassion fatigue “Loving-kindness meditation” (a state of self-awareness and repeating phrases to reinforce a positive attitude with compassion for oneself and others) At least 3 times a week App (Whatsapp, where audio files for the meditation were distributed) Participants were organized into 2 WhatsApp groups for interaction with the researchers and fellow participants N/A^e^	Miscellaneous files on mental health during the COVID-19 pandemic period	Post 6 week	MH^f^ symptoms and disorders: N/A Emotional exhaustion and compassion fatigue compassion fatigue (NCFI^g^) Positive MH and well-being: N/A	N/A^h^
Hwang and Jo [40], 2019	2018	South Korea	Nurses employed at college hospitals (N=56)	4 weeks Stress management Meditation, breathing methods, and yoga More than twice per week for at least 10 minutes each session App N/A Music focused on healing, health information for mental health care every week (diet, benefits of exercise, and so on)	Waitlist	Post 4 week	MH symptoms and disorders: Perceived stress (PSS-10), depression (PHQ^i^-9), anxiety (GAD-7) Emotional exhaustion and compassion fatigue: N/A Positive MH and well-being: well-being (WHO-5^j^), self-efficacy (Likert scale)	N/A
Keng et al [41], 2022	No information but under COVID-19 pandemic	Singapore	Health care workers (N=80)	1.5 weeks Reduction of mental health problems during the COVID-19 pandemic Mindful breathing, mindfulness of thoughts, and mindfulness of sounds 10 minutes per day App (“Headspace") N/A N/A	Active control (“Lumosity” a daily training component of 3 free games involving problem solving, memory, and attention, totaling 10 minutes, with a 3-week daily practice)	Post 3 weeks FU 4 weeks	MH symptoms and disorders: depression and anxiety (DASS^k^-21), PTSD^l^ symptoms (PCL-C^m^), fear of COVID-19 (FCV-19S^n^), sleep quality (PSQI^o^) Emotional exhaustion and compassion fatigue: N/A Positive MH and well-being: Personal well-being (PWI^p^)	56.41% (22/39) completed ≥80%, 30.76% completed 40%‐80%, and 12.8% completed 20%‐40% of the required practice duration
Lilly et al [42], 2019	2016-2017	United States or Canada	Emergency medical dispatchers (telecommunicators; N=323)	7 weeks Reduction of work-related stress for emergency medical dispatchers 7 modules MBSR^q^-based short videos, text descriptions, and audio-guided meditation exercises 20‐30 minutes per module per week, with additional daily practice encouraged (5‐10 min) Web (“Destress 9-1-1”) Moderated discussion board N/A	Waitlist	Post 7 weeks FU 12 weeks	MH symptoms and disorders: stress symptoms (C-SOSI^r^) Emotional exhaustion and compassion fatigue: N/A Positive MH and well-being: N/A	89/161 (55.3%) completed 6 or 7 modules. Mean number of days/wk: 2.1 (SD=1.5, range 0‐6.8).
Luangapichart et al [43], 2022	2021	Thailand	Medical personnel with moderate or high levels of burnout and stress from across Thailand (N=90)	4 weeks Reduction of burnout and stress Mindfulness (by using body sensations, surrounding sounds, and front images) At least 3 times daily App (“Mindful Senses” an online audio-based program via the LINE application) Participants were able to send inquiries regarding mindfulness practice to the therapist via the LINE application at any time during the program and received a reply within 1 day Psychological self-help articles (PSAs) on burnout, stress management, relationship management, and mental health promotion	Active control (PSA only)	Post 4 weeks FU 4 weeks	MH symptoms and disorders: stress (ST^s^-5), anxiety (HADS^t^), and depression (HADS) Emotional exhaustion and compassion fatigue: N/A Positive MH and well-being: N/A	22.2% listened to the audio files more than 3 times per day for over 60% of the total 4 weeks.
Nourian et al [44], 2021	2020	Iran	Nurses working in the COVID-19 care units (N=41)	7 weeks Reduction of stress MBSR, including meditations and yoga exercises At least 1 hour per day, 6 days a week App (WhatsApp Messenger) Group and individual guidance was provided through 2-person chats and short messages to resolve any issues related to the exercises for that week N/A	Music ﬁles or training items on caring for patients with COVID-19	Post 7 weeks	MH symptoms and disorders: sleep quality (PSQI) Emotional exhaustion and compassion fatigue: N/A Positive MH and well-being: N/A	N/A
Pratt et al [45], 2023	2021-2022	United States	Nurses working in COVID-19 units at a single hospital (N=102)	4 weeks Reduction of psychological distress and burnout Mindfulness (“Lift”) Daily Mobile app If participants reported suicidal ideation, they were immediately contacted by phone and connected to mental health service providers N/A	Waitlist	Post 4 weeks	MH symptoms and disorders: depression (PHQ-9), anxiety (GAD^u^-7), and stress (PSS-4) Emotional exhaustion and compassion fatigue Emotional exhaustion (MBI^v^) Positive MH and well-being: N/A	27.5% completed at least one mindfulness session per week, 18.8% completed ≥75% of mindfulness sessions
Taylor et al [46], 2022	2017-2018	England	National Health Service staff (N=2182)	18 weeks Reduction of stress, mental health, and work-related outcomes Brief mindfulness-based practices alongside psychoeducational materials 4. Completing the Take 10 introductory pack (guided 10-min mindfulness practices daily for 10 consecutive days) and at least one 10-minute mindfulness practice daily for the duration of the study Website or App (“Headspace") N/A N/A	Active control (“NHS^w^ Moodzone,” a psychoeducational digital platform offering evidence-based advice and resources on managing work-related stress and mental health, including videos, audio, and podcasts, with 10 min daily)	Post 18 weeks	MH symptoms and disorders: depression, anxiety, and stress (DASS) Emotional exhaustion and compassion fatigue Emotional exhaustion (MBI) Positive MH and well-being: Well-being (SWEMWBS^x^)	Average 3.5 days per week; 66.6% practiced at least 3 days a week
Xu et al [47], 2022	2019-2020	Australia	Staff in 2 emergency departments (N=148)	4 weeks Reduction of emergency department work stress Guided mindfulness meditation with access to over 50 sessions ranging from 3 to 20 minutes Daily practice of 10 minutes, with the option to choose preferred sessions App (“Headspace") N/A N/A	Waitlist	Post 4 weeks FU 12 weeks	MH symptoms and disorders: stress (PSS^y^); emotional exhaustion and compassion fatigue: emotional exhaustion (MBI); positive MH and well-being: well-being (WEMWBS^z^)	Most frequent use was 1‐3 times per week (41%) of 2‐10 min sessions (68%)

a All studies allocated participants to the intervention group and the control group in a 1:1 ratio.

b (1) Intervention period, (2) program objective, (3) content, (4) intensity, (5) platform, (6) interpersonal support, and (7) other elements besides mindfulness.

c FU: follow-up.

d NICU: neonatal intensive care unit.

e N/A: not assessed.

f MH: mental health.

g NCFI: Nurses' Compassion Fatigue Inventory.

h Not applicable.

i PHQ: Patient Health Questionnaire.

j WHO-5: World Health Organization-5 Well-Being Index.

k DASS: Depression Anxiety Stress Scales.

l PTSD: posttraumatic stress disorder.

m PCL-C: Post-Traumatic Stress Disorder Checklist–Civilian Scale.

n FCV-19S: Fear of COVID-19 Scale.

o PSQI: Pittsburgh Sleep Quality Index.

p PWI: Personal Wellbeing Index.

q MBSR: mindfulness-based stress reduction.

r C-SOSI: Calgary Symptoms of Stress Inventory.

s ST: Stress Test Questionnaire.

t HADS: Hospital Anxiety and Depression Scale.

u GAD: Generalized Anxiety Disorder.

v MBI: Maslach Burnout Inventory.

w NHS: National Health Service.

x SWEMWBS: Short Warwick Edinburgh Mental Well-Being Scale.

y PSS: Perceived Stress Scale.

z WEMWBS: Warwick Edinburgh Mental Well-Being Scale.

### Characteristics of Interventions

The average intervention period was 6.17 weeks, ranging from 1.5 weeks [41] to 18 weeks [46]. Regarding the platforms used:

- Commercial apps: three RCTs [41,46,47] used interventions involving a commercial mindfulness app (“Headspace”).
- Social networking service (SNS) tools: three RCTs [39,43,44] used interventions involving SNS messaging platforms.
- Other platforms: others used original apps [40,45] and a web-based program [42].

Regarding follow-up assessments, 5 studies [39,40,44-46] measured outcomes only immediately postintervention, and 4 [41-43,47] measured outcomes twice: immediately postintervention and at follow-up (ranging from 4 to 12 wk after the postintervention period). No studies evaluated long-term effectiveness beyond this timeframe.

### Outcome Measures and Data Synthesis

The included studies assessed a broad range of psychological outcomes. Perceived stress was the most frequently measured outcome (n=6) [40,42,43,45-47], followed by depression and anxiety (n=5) [40,41,43,45,46]. Other measures included emotional exhaustion and compassion fatigue (n=4) [39,45-47], as well as positive mental health constructs such as well-being (n=4) [40,41,46,47].

Certain outcomes, including sleep quality (n=2) [41,44], self-efficacy (n=1) [40], symptoms of PTSD (n=1) [41], and fear of COVID-19 (n=1) [41], were excluded from the meta-analysis due to an insufficient number of studies for robust synthesis.

Adherence to the prescribed dose was reported in 6 out of 9 studies. Program completion rates were documented in 3 studies [41,42,45]. Among these, 56.4% (22/39) of participants completed more than 80% of the required practice duration in one study [41], and 55.3% (89/161) completed 6 or 7 modules in another [42]. In contrast, 1 study reported that 19% (13/69) of participants completed more than 75% of the daily sessions [45]. The average frequency of weekly usage was reported in 5 studies [42,43,45-47]. Specifically, 28% (19/69) of participants completed at least 1 session per week [45], 41% (29/71) used the program 1 to 3 times per week [47], and the average weekly usage was 2.1 (SD 1.5) days [42]. In the Taylor et al [46] study, 66.6% (452/679) of participants practiced 3 or more days per week. Furthermore, in the Luangapichart et al [43] study, 22.2% (10/45) of participants used the program 4 or more days per week, with 3 or more sessions per day.

### Risk of Bias

Table 2 presents the risk of bias assessed using RoB 2.0. All studies were evaluated as having a high risk of bias. In many studies, domains 1, 2, and 5 were rated as low or as having some concerns; however, domains 3 and 4 were frequently rated as high. Consequently, the overall bias across all studies was determined to be high.

**Table 2.** Summary of the risk of bias assessment for the included studies.

First author, year	Domain 1 (R^a^)	Domain 2 (D^b^)	Domain 3 (Mi^c^)	Domain 4 (Me^d^)	Domain 5 (S^e^)	Overall
Outcome: depression (meta-analysis conducted)						
Hwang and Jo [40], 2019	?	+^f^	–	+	^?g^	–^h^
Keng et al [41], 2022	?	?	+	–	?	–
Luangapichart et al [43], 2022	+	?	+	–	+	–
Pratt et al [45], 2023	?	?	–	–	+	–
Taylor et al [46], 2022	+	?	–	–	?	–
Outcome: anxiety (meta-analysis conducted)						
Hwang and Jo [40], 2019	?	+	–	+	?	–
Keng et al [41], 2022	?	?	+	–	?	–
Luangapichart et al [43], 2022	+	?	+	–	+	–
Pratt et al [45], 2023	?	?	–	–	+	–
Taylor et al [46], 2022	+	?	–	–	?	–
Outcome: perceived stress (meta-analysis conducted)						
Hwang and Jo [40], 2019	?	+	–	+	?	–
Lilly et al [42], 2019	?	?	–	–	?	–
Luangapichart et al [43], 2022	+	?	+	–	+	–
Pratt et al [45], 2023	?	?	–	–	+	–
Taylor et al [46], 2022	+	?	–	–	?	–
Xu et al [47], 2022	+	?		–	+	–
Outcome: emotional exhaustion and compassion fatigue (meta-analysis conducted)						
Asadollah et al [39], 2023	–	–	+	–	?	–
Pratt et al [45], 2023	?	?	–	–	+	–
Taylor et al [46], 2022	+	?	–	–	?	–
Xu et al [47], 2022	+	?	–	–		–
Outcome: positive mental health and well-being (meta-analysis conducted)						
Hwang and Jo [40], 2019	?	+	–	+	?	–
Keng et al [41], 2022	?	?	+	–	?	–
Taylor et al [46], 2022	+	?	–	–	?	–
Xu et al [47], 2022	+	?	–	–	+	–
Outcome: PTSD (meta-analysis not conducted)						
Keng et al [41], 2022	?	?	+	–	?	–
Outcome: fear of COVID-19 (meta-analysis not conducted)						
Keng et al [41], 2022	?	?	+	–	?	–
Outcome: sleep quality (meta-analysis not conducted)						
Keng et al [41], 2022	?	?	+	–	?	–
Nourian et al [44], 2021	?	–	–	–	?	–
Outcome: self-efficacy (meta-analysis not conducted)						
Hwang and Jo [40], 2019	?	+	–	+	?	–

a R: bias arising from the randomization process.

b D: bias due to deviations from intended interventions.

c Mi: bias due to missing outcome data.

d Me: bias in measurement of the outcome.

e S: bias in selection of the reported result.

f +: low risk of bias.

g ?: some concerns.

h -: high risk of bias.

### Meta-Analysis

**Figure 2.**
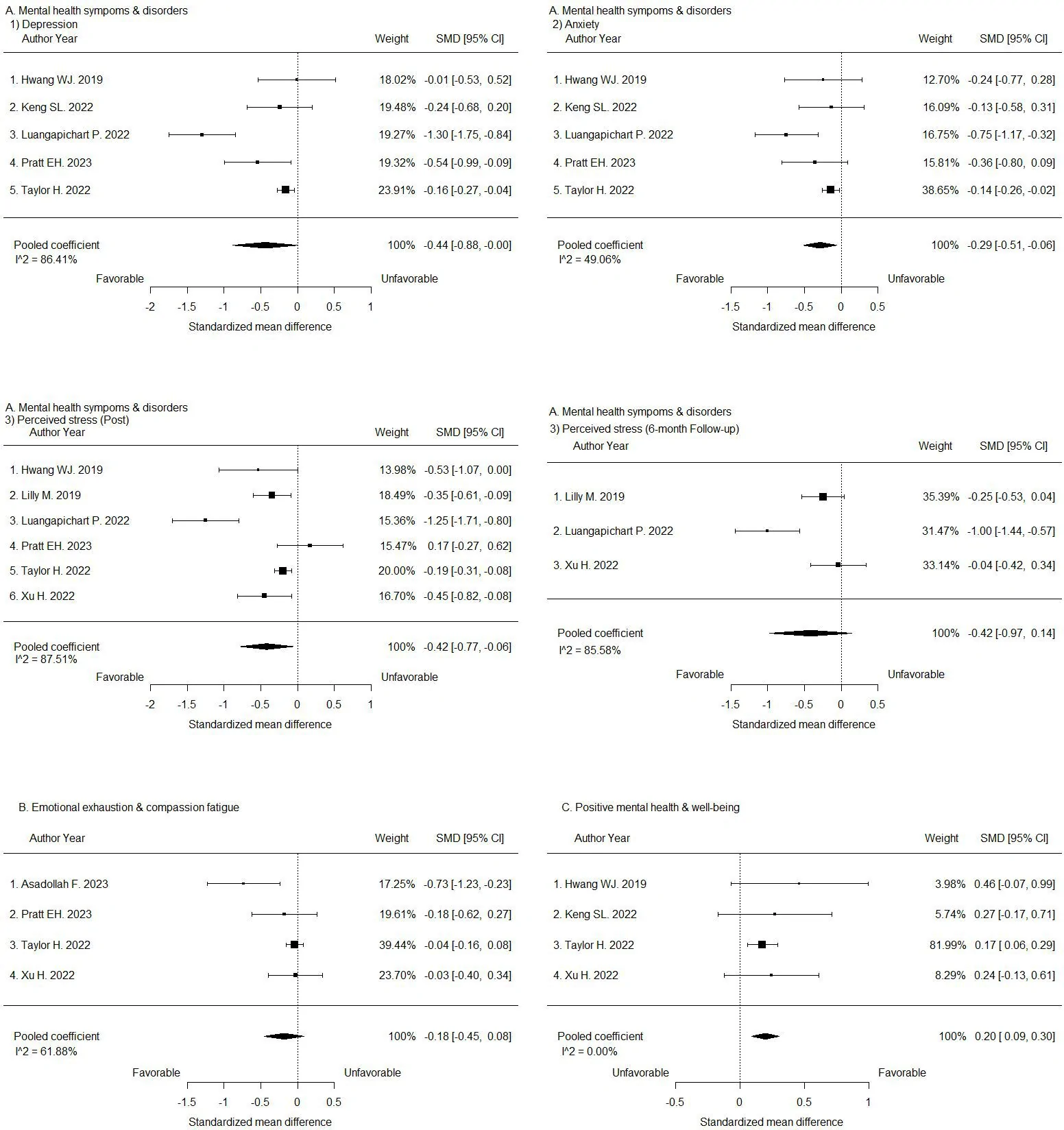
Summary of meta-analysis [39-43,45-47]. (A) Mental health symptoms and disorders: (1) depression and (2) anxiety [40, 41, 43, 45, 46], (3) perceived stress (post) [40, 42, 43, 45, 46, 47], (4) perceived stress (6-mo follow-up) [42, 43, 47]. (B) Emotional exhaustion and compassion fatigue [39, 45, 46, 47]. (C) Positive mental health and well-being [40, 41, 46, 47].

The results of the meta-analysis are shown in Figure 2. Digital mindfulness interventions demonstrated significant beneficial effects in improving the postintervention scores of individuals with depression (k=5, SMD=-0.44, 95% CI −0.88 to −0.003; *P*=.049), anxiety (k=5; SMD=−0.29, 95% CI −0.51 to −0.06; *P*=.011), perceived stress (k=6; SMD=−0.42, 95% CI −0.77 to −0.06; *P*=.021), and well-being (k=4; SMD=0.20, 95% CI 0.09 to 0.30; *P*<.001). No significant effectiveness was observed in addressing emotional exhaustion or compassion fatigue. Additionally, no long-term efficacy was identified for perceived stress during the follow-up periods, which ranged from 4 to 12 weeks after the postintervention period. The heterogeneity varied from low to high (*I*^2^=0% in well-being, 50%‐60% in anxiety and emotional exhaustion or compassion fatigue, and over 80% in depression and perceived stress).

Sensitivity analyses excluding Luangapichart et al [43], which reported a notably larger intervention effect, significantly reduced heterogeneity for depression (k=4; SMD=−0.18, 95%CI −0.28 to −0.07; *P*=.001; *I*^2^=0%) and postintervention perceived stress (k=5; SMD=−0.25, 95% CI −0.40 to −0.10; *P*<.001; *I*^2^=28.1%).

### Publication Bias

Multimedia Appendix 3 presents funnel plots of the included studies. We qualitatively and quantitatively assessed publication bias by visually inspecting funnel plot asymmetry. The visual inspection suggested symmetry. Egger tests were not significant for all outcomes. Although no clear evidence of funnel plot asymmetry was observed, these findings should be interpreted cautiously due to the limited number of included studies.

### Certainty of Evidence

The GRADE evidence profile is presented in Multimedia Appendix 4. The certainty of evidence for the outcomes included in the analysis ranged from “Low” to “Very Low.”

## Discussion

### Principal Findings

#### Overview

This systematic review and meta-analysis evaluated fully automated digital mindfulness interventions for HCWs, ultimately including 9 RCTs with 3088 participants. Adherence and dose varied widely across the included studies. Results showed that these interventions had moderate, beneficial effects on depression (SMD=−0.44, 95% CI −0.88 to −0.003), anxiety (SMD=−0.29, 95% CI −0.51 to −0.06), and perceived stress (SMD=−0.42, 95% CI −0.77 to −0.06) immediately postintervention, as well as a slight improvement in well-being (SMD=0.20). However, the interventions did not significantly impact emotional exhaustion or compassion fatigue, and there was no evidence of sustained effects on perceived stress during the follow-up period, which ranged from 4 to 12 weeks after the postintervention period. Heterogeneity across studies was low in well-being but moderate to high in other outcomes.

#### Effects on Depression, Anxiety, and Perceived Stress

Digital mindfulness intervention was effective in improving depression, anxiety, and perceived stress among HCWs. This result was consistent with a previous report that workplace mindfulness interventions improved mental health [9]. While previous meta-analyses of workplace mindfulness interventions, which predominantly included face-to-face or facilitator-guided programs, reported large pooled effects on composite mental health outcomes (eg, SMDs >0.6) [9], the present review demonstrated moderate effects (eg, SMD=−0.44, 95% CI −0.88 to −0.003 for depression) despite focusing exclusively on self-guided digital interventions. This pattern is consistent with the lower intensity of digital interventions. An earlier systematic review reported the effectiveness of web-based mindfulness interventions, including those with professional assistance for HCWs during COVID-19 [18]. However, it included an app with various components (ie, cognitive behavioral approach) other than mindfulness [48] and half of the included studies were from one specific country, leading to a risk of overestimation of effectiveness. In this study, we excluded interventions that did not focus solely on mindfulness or those with professional or personal support. The present findings could provide more precise insights into the effects of digital mindfulness interventions as a primary prevention strategy for the mental health of HCWs.

#### Effects on Well-Being and Potential Mechanisms

The results demonstrated the effectiveness in improving the well-being of HCWs. This finding was consistent with previous reviews [9,12]. Our review measured well-being using the World Health Organization-Five Well-Being Index (WHO-5) and the Warwick Edinburgh Mental Well-being Scale (WEMWBS). Mindfulness enhances skills to accept negative emotions and to cope with distress [49,50], leading to emotional stability and calmness. Additionally, mindfulness promotes greater attention to and awareness of present moment experiences [27] and may encourage individuals to be active and vigorous in pursuing something that they want to focus on. Positive affects, including relaxed, calm, active, vigorous, and energetic states, were measured by both WHO-5 and WEMWBS. Mindfulness also nurtures self-acceptance and self-compassion; it may reduce self-criticism and improve self-esteem. This contributes to enhancing items on the WEMWBS, such as “feeling good about myself” and “feeling confident.” Digital mindfulness interventions can increase positive aspects of mental health while also reducing negative emotions.

#### Effects on Emotional Exhaustion and Compassion Fatigue

No significant effectiveness was shown in addressing emotional exhaustion or compassion fatigue. One previous systematic review of mindfulness for nurses reported its effectiveness in improving personal accomplishment (a subscale of burnout), but no effectiveness was observed for emotional exhaustion or depersonalization [51]. Another meta-analysis of interventions for nurses also reported nonsignificant effectiveness across all subscales of burnout [52]. However, these meta-analyses included only 2 studies to estimate the efficacy. More studies are needed to draw firm conclusions. A previous review of RCTs of both individual-based and organization-based interventions for reducing burnout among physicians reported beneficial integrated effectiveness on all burnout subscales [53]. There remains uncertainty about the effectiveness of digital mindfulness interventions on emotional exhaustion. Nevertheless, it may be necessary to consider using such interventions in conjunction with structural or organizational strategies (eg, working hour limitation)

#### Cultural and Occupational Contexts

Cultural and occupational contexts may also influence the effectiveness of digital mindfulness interventions. The included RCTs were conducted across diverse regions, including Europe and Asia, where cultural norms regarding emotional expression, self-care, and help-seeking may shape engagement with mindfulness practices. In addition, occupational differences among HCWs, such as those between nurses and physicians, may affect baseline stressors, workload, and responsiveness to self-guided interventions. These contextual factors should be considered when interpreting the findings and their generalizability.

#### Heterogeneity Across Interventions

A substantial heterogeneity observed in this study may also be attributed to the variability in intervention characteristics, including duration, number of modules, and delivery modes. For instance, intervention durations ranged from 1.5 weeks [41] to 18 weeks [46], and content delivery varied between smartphone apps (eg, commercial apps [41,46,47], original apps [40,45], SNS tools [39,43,44]), and web-based platforms [42]. Although the limited number of studies precluded a subgroup analysis to statistically quantify the impact of these variations, our sensitivity analysis indicated that excluding specific outliers significantly reduced statistical heterogeneity. This suggests that while structural differences exist, they may not be the sole driver of the observed inconsistency. Nevertheless, this variability highlights a challenge in synthesizing evidence. To facilitate more precise comparisons and identify optimal intervention components, future research should strive for greater standardization in intervention protocols and reporting.

### Limitations

While our meta-analysis indicated a beneficial trend of the intervention on mental health outcomes, the certainty of evidence assessed by the GRADE approach remained “Low” or “Very Low.” These results suggest that the true effect may be substantially different from the estimate of the effect. The primary factor reducing the certainty was the “very serious risk of bias” identified in all included studies. Methodological limitations, such as a lack of blinding or allocation concealment and reliance on self-reported outcomes, were present in many studies and may have led to an overestimation of the effects. Furthermore, inconsistency across studies for depression and perceived stress outcomes, as well as imprecision of the estimates for emotional exhaustion, further contributed to the lower certainty grades. Therefore, the current findings should be interpreted with caution. To establish more certain evidence, future research requires well-designed, high-quality RCTs that minimize the risk of bias. In addition to these points, this review has several limitations. First, only nine studies met the inclusion criteria, which restricts the generalizability of the findings. The inclusion of outcome-related terms in the search strategy may have increased the risk of missing some relevant studies, as noted in the Cochrane Handbook [25], and should be considered a limitation of this review. Additionally, a high risk of bias was observed in the quality assessment using the RoB2 tool, primarily due to self-reported outcome measures. Due to the nature of digital mindfulness interventions as psychosocial interventions, blinding of participants was not feasible in most studies, which may have introduced performance bias alongside reporting and detection bias. This introduces potential bias and may impact the reliability of the results. Furthermore, the included studies exhibit considerable heterogeneity in intervention content, making it challenging to draw consistent conclusions across studies. In addition, the quality of the included digital mindfulness applications may have varied, as not all interventions were clearly reported to be developed or supervised by qualified mindfulness experts. This variability in app quality should be considered when interpreting the findings. The review also includes several RCTs with small sample sizes, limiting the statistical power of these studies. The results from Egger tests and a visual inspection of the funnel plots provide little indication of publication bias. Yet, because only a small number of studies were included, conclusions regarding publication bias should be drawn with caution. The language restriction to studies published in English or Japanese may have resulted in the exclusion of relevant research published in other languages, thereby narrowing the scope of this review. The long-term effects of the interventions still need to be clarified, as few studies evaluated outcomes over an extended period. Adherence rates were reported in some studies but could not be formally linked to outcomes due to limited and inconsistent reporting. Future individual participant data meta-analyses could examine adherence–outcome relationships in self-guided digital mindfulness interventions. Although classified as self-guided, some interventions allowed minimal asynchronous contact, which may have influenced adherence and effect size. Finally, this study’s findings, derived from RCTs, present a limitation because the inherent research design often involves human-mediated interventions, such as reminders and economic rewards, which likely enhance adherence. Therefore, in real-world settings where such self-guided digital mindfulness interventions are used without human facilitation, the opportunity to achieve similar levels of adherence—and thereby comparable effects—may be significantly reduced, potentially resulting in smaller observed effects in real-world data contexts. Although beyond the primary scope of this review, ethical and implementation considerations such as user privacy, data security, and responsible data management are important issues for the real-world deployment of digital mindfulness interventions. These factors should be carefully addressed in future research and implementation efforts.

### Conclusions

Given the high risk of bias across all included studies, the findings should be interpreted with caution. Nevertheless, health care organizations may consider integrating digital mindfulness interventions as an accessible, short-term mental health support tool for reducing depression, anxiety, and perceived stress among HCWs. However, additional strategies may be needed to address persistent emotional exhaustion and compassion fatigue.

## Supplementary material

10.2196/74272 Multimedia Appendix 1 Search terms.

10.2196/74272 Multimedia Appendix 2 Excluded studies with reasons.

10.2196/74272 Multimedia Appendix 3 Results of funnel plots of the included studies.

10.2196/74272 Multimedia Appendix 4 GRADE Evidence profile.

10.2196/74272 Checklist 1 PRISMA checklist.

## Abbreviations

GRADE: Grading of Recommendations Assessment, Development and Evaluation
HCW: health care worker
PICO: participants, interventions, comparisons, and outcomes
PRISMA: Preferred Reporting Items for Systematic Review and Meta-Analysis
RCT: randomized controlled trial
SMD: standardized mean difference
SNS: social networking service
WEMWBS: Warwick Edinburgh Mental Well-being Scale
WHO-5: World Health Organization-Five Well-Being Index
