# Supplementary material for: Effects of Self-Guided Digital Mindfulness Interventions on the Mental Health of Health Care Workers: Systematic Review and Meta-Analysis of Randomized Controlled Trials
Source: JMIR Mhealth Uhealth. 2026 Aug 21;14:e74272. doi: 10.2196/74272 (PMC13494152; doi:10.2196/74272)
Supplement: Supplementary file 2 [file mhealth-v14-e74272-s001.docx]

**Supplementary file 1. Search terms.**

**PubMed**

("Occupational Groups"[Mesh] OR "Occupational Health"[Mesh] OR "enterprise*"[tiab] OR "business*"[tiab] OR "employed"[tiab] OR "employee*"[tiab] OR "employer*"[tiab] OR "employment*"[tiab] OR "informal sector*"[tiab] OR "informal work*"[tiab] OR "laborer*"[tiab] OR "labourer*"[tiab] OR "occupation*"[tiab] OR "personnel*"[tiab] OR "professional"[tiab] OR "professionals"[tiab] OR "staff"[tiab] OR "staffing*"[tiab] OR "vocation"[tiab] OR "vocations"[tiab] OR "worker"[tiab] OR "workers"[tiab] OR "workforce"[tiab] OR "workplace*"[tiab] OR "work place*"[tiab] OR "worksite*"[tiab] OR "work site*"[tiab] OR "aide"[tiab] OR "aides"[tiab] OR "audiologist*"[tiab] OR "ambulance*"[tiab] OR "care assistant*"[tiab] OR "clinician*"[tiab] OR "coastguard*"[tiab] OR "coast guard*"[tiab] OR "dentist*"[tiab] OR "detective*"[tiab] OR "disaster responder*"[tiab] OR "doctor*"[tiab] OR "domestic health care"[tiab] OR "domiciliary care*"[tiab] OR "emergency service*"[tiab] OR "emergency responder*"[tiab] OR "emergency medical"[tiab] OR "firefighter*"[tiab] OR "fire fighter*"[tiab] OR "first responder*"[tiab] OR "general practitioner*"[tiab] OR "health care provider*"[tiab] OR "healthcare provider*"[tiab] OR "health visitor*"[tiab] OR "home care service*"[tiab] OR "humanitarian aid*"[tiab] OR "humanitarian relie*"[tiab] OR "humanitarian service*"[tiab] OR "law enforc*"[tiab] OR "lifeguard*"[tiab] OR "life guard*"[tiab] OR "medical resident*"[tiab] OR "medic"[tiab] OR "medics"[tiab] OR "nurse*"[tiab] OR "nursing"[tiab] OR "midwi*"[tiab] OR "paramedic*"[tiab] OR "policemen"[tiab] OR "policeman"[tiab] OR "police men"[tiab] OR "police man"[tiab] OR "police women"[tiab] OR "police woman"[tiab] OR "police officer*"[tiab] OR "firemen"[tiab] OR "fireman"[tiab] OR "fire men"[tiab] OR "fire man"[tiab] OR "fire women"[tiab] OR "fire woman"[tiab] OR "pharmacis*"[tiab] OR "psychologist*"[tiab] OR "physician*"[tiab] OR "practitioner*"[tiab] OR "relief work*"[tiab] OR "rescuer*"[tiab] OR "rescue work*"[tiab] OR "therapist*"[tiab] OR "veterinaria*"[tiab])

AND

(((randomized[tiab] AND trial*[tiab]) OR (randomised[tiab] AND trial*[tiab]) OR "randomized control*"[tiab] OR "randomised control*"[tiab] OR "randomised design*"[tiab] OR "randomized design*"[tiab]) NOT (“letter”[pt] OR “comment”[pt] OR “case report”[tw] OR “editorial”[pt] OR "Meta-Analysis"[PT] OR "Systematic Review"[PT] OR (("Animals"[Mesh] OR "Models, Animal"[Mesh]) NOT humans[mh])))

AND

((online*[tiab] OR internet*[tiab] OR web[tiab] OR app[tiab] OR apps[tiab] OR computer*[tiab] OR digital*[tiab] OR smartphone*[tiab] OR game*[tiab] OR telemedicine*[tiab] OR smart-phone*[tiab] OR e-health[tiab] OR ehealth[tiab] OR mobile*[tiab] OR mhealth[tiab] OR m-health[tiab] OR wearable*[tiab] OR "smartwatch"[tiab] OR "smart-watch"[tiab] OR “biosensing”[tiab] OR “wearable electronic device”[tiab] OR “mobile app”[tiab] OR "contactless"[tiab] OR electroencephalogra*[tiab] OR "magnetic resonance imaging"[tiab] OR "neurofeedback"[Mesh] OR "brain computer interfaces"[Mesh] OR "fNIRS"[tiab] OR "alpha wave"[tiab] OR "EEG"[tiab] OR arrhythmias[Mesh] OR "cardiac output"[tiab] OR "photoplethysmography"[tiab] OR "PPG"[tiab] OR electrocardiogra*[tiab] OR "ECG"[tiab] OR "RR interval"[tiab] OR "LF/HF"[tiab] OR "oxygen saturation"[tiab] OR "heart rate determination"[tiab] OR "rPPG"[tiab] OR "robotics"[tiab] OR "robot therapy"[tiab] OR "robot assisted therapy"[tiab] OR "chatbot"[tiab] OR "chatGPT"[tiab] OR “Virtual Reality”[tiab] OR "VR"[tiab] OR "internet based intervention"[tiab] OR "metaverse"[tiab] OR "facial recognition"[tiab] OR "voice recognition"[tiab] OR "wavelet analysis"[tiab] OR "speech recognition software"[tiab] OR "phonetics"[tiab] OR "natural language processing"[tiab] OR "machine learning"[tiab] OR "artificial intelligence"[tiab] OR "deep learning"[tiab] OR "data mining"[tiab] OR “neural networks”[tiab] OR “signal processing”[tiab] OR "speech analysis"[tiab] OR "voice analysis"[tiab] OR "physiological signals"[tiab] OR "speech synthesis"[tiab] OR "motion capture"[tiab] OR "fitness trackers"[tiab] OR "accelerometry"[tiab] OR "actigraphy"[tiab] OR "biomechanical phenomena"[tiab] OR "ecological parameter monitoring"[tiab] OR electromyogra*[tiab] OR “motion sensor”[tiab] OR "gyroscope"[tiab] OR "polysomnography"[Mesh] OR "sleep stages"[Mesh] OR "oximetry"[Mesh] OR "snoring"[Mesh] OR electrooculogra*[tiab] OR "heart rate variability"[tiab] OR "HRV"[tiab] OR "sleep efficiency"[tiab] OR "PSG"[tiab] OR "digital psychotherapy"[tiab] OR "thermometers"[tiab] OR "eye tracking"[tiab] OR "ultrasonics"[tiab] OR "photoacoustic techniques"[tiab] OR "photometry"[tiab] OR “text mining”[tiab] OR “functional MRI”[tiab] OR “fMRI”[tiab] OR “heart rhythm”[tiab] OR ”heart rate monitoring”[tiab] OR “socially assistive robot”[tiab] OR “facial expression recognition”[tiab] OR “activity tracker”[tiab] OR “biomechanics”[tiab] OR “pulse oximeter”[tiab] OR “SpO2”[tiab]) OR ((online*[tiab] OR internet*[tiab] OR web[tiab] OR app[tiab] OR apps[tiab] OR computer*[tiab] OR digital*[tiab] OR smartphone*[tiab] OR game*[tiab] OR telemedicine*[tiab] OR smart-phone*[tiab] OR e-health[tiab] OR ehealth[tiab] OR mobile*[tiab] OR mhealth[tiab] OR m-health[tiab] OR wearable*[tiab] OR "smartwatch"[tiab] OR "smart-watch"[tiab] OR “biosensing”[tiab] OR “wearable electronic device”[tiab] OR “mobile app”[tiab]) AND ("heart rate"[tiab] OR "respiration"[tiab] OR "blood pressure"[tiab] OR "exercise"[tiab] OR "body temperature"[tiab] OR "sedentary behavior"[tiab] OR "walking"[tiab] OR "sleep"[tiab] OR "diet"[tiab] OR meal*[tiab] OR "nutritional status"[tiab] OR "eating"[tiab] OR "energy intake"[tiab] OR "feeding behavior"[tiab] OR "food intake"[tiab] OR "crew resource management"[tiab] OR "mentoring"[tiab] OR "psychological safety"[tiab] OR "stress management training"[tiab] OR "teamwork training"[tiab] OR "communication training"[tiab] OR "cognitive behavioral therapy"[tiab] OR "play therapy"[tiab] OR "cognitive training"[tiab] OR "mindfulness"[tiab] OR "resistance"[tiab] OR “physical activity”[tiab] OR "sedentary time"[tiab] OR “eating behavior”[tiab] OR “music therapy”[tiab])))

AND

("Optimism"[Mesh] OR "Personal Satisfaction"[Mesh] OR "Self Concept"[Mesh:NoExp] OR "Self Efficacy"[Mesh] OR "Self-Control"[Mesh] OR "life engag*"[tiab] OR "life satisf*"[tiab] OR "meaning of life"[tiab] OR "purpose in life"[tiab] OR "positive affect*"[tiab] OR "positive emotion*"[tiab] OR "resilien*"[tiab] OR "self concept*"[tiab] OR "self control*"[tiab] OR "self efficac*"[tiab] OR "self esteem*"[tiab] OR "swb"[tiab] OR "well being*"[tiab] OR "wellbeing*"[tiab] OR "Mental Disorders"[Mesh] OR "Mental Health"[Mesh] OR "Psychology, Industrial"[Mesh] OR "Stress, Psychological"[Mesh] OR "adjustment"[tiab] OR "affective disorder*"[tiab] OR "anxiet*"[tiab] OR "bipolar*"[tiab] OR "burn out*"[tiab] OR "burnout*"[tiab] OR "CMD" [tiab] OR "depressi*"[tiab] OR "eating disorder*"[tiab] OR "mental disorder*"[tiab] OR "mental health*"[tiab] OR "mental illness*"[tiab] OR "mood disorder*"[tiab] OR "obsessive compulsive disorder*"[tiab] OR "ocd"[tiab] OR "panic disorder*"[tiab] OR "phobi*"[tiab] OR "post traumatic*"[tiab] OR "psychiatric diagnos*"[tiab] OR "psychiatric disease*"[tiab] OR "psychiatric disorder*"[tiab] OR "psychiatric illness*"[tiab] OR "psychological disorder*"[tiab] OR "psychos*"[tiab] OR "psychotic*"[tiab] OR "psychological distress*"[tiab] OR "ptsd"[tiab] OR "ptss"[tiab] OR "somatoform disorder*"[tiab] OR "schizophren*"[tiab] OR "stress*"[tiab] OR "Self-Injurious Behavior"[Mesh] OR "auto mutilat*"[tiab] OR "automutilat*"[tiab] OR "intentional injur*"[tiab] OR "self harm*"[tiab] OR "selfharm*"[tiab] OR "suicid*"[tiab] OR "Substance-Related Disorders"[Mesh] OR "alcohol abus*"[tiab] OR "alcohol misus*"[tiab] OR "alcohol us*"[tiab] OR "alcoholis*"[tiab] OR "amphetamin*"[tiab] OR "binge drinking*"[tiab] OR "cannabis*"[tiab] OR "cocain*"[tiab] OR "diacetylmorphin*"[tiab] OR "diamorphin*"[tiab] OR "drug abus*"[tiab] OR "drug misus*"[tiab] OR "drug us*"[tiab] OR "drunk*"[tiab] OR "drinking"[tiab] OR "ecstasy*"[tiab] OR "xtc"[tiab] OR "fentanyl*"[tiab] OR "hashish*"[tiab] OR "heroin*"[tiab] OR "marihuan*"[tiab] OR "marijuan*"[tiab] OR "mdma"[tiab] OR "methadon*"[tiab] OR "methamphetamin*"[tiab] OR "methylenedioxymethamphetamin*"[tiab] OR "morphin*"[tiab] OR "opiate*"[tiab] OR "opioid*"[tiab] OR "thc"[tiab] OR "crystal meth*"[tiab] OR "Quality of Life"[Mesh] OR "Quality-Adjusted Life Years"[Mesh] OR "disability adjusted life"[tiab] OR "qaly"[tiab] OR "daly"[tiab] OR "functioning"[tiab] OR "functional abilit*" OR "functionalit*" OR "hrqol*"[tiab] OR "life activit*"[tiab] OR "life participati*"[tiab] OR "life stress*"[tiab] OR "qol"[tiab] OR "qoli"[tiab] OR "quality of life*"[tiab] OR "self car*"[tiab] OR "selfcar*"[tiab] OR "sickness impact profile*" [tiab] OR "social function*"[tiab] OR "social participati*"[tiab] OR "Absenteeism"[Mesh] OR "Employment"[Mesh] OR "Job Satisfaction"[MeSH] OR "Sick Leave"[Mesh] OR "Retirement"[Mesh] OR "Work Capacity Evaluation"[Mesh] OR "absenteeism*"[tiab] OR "back to work"[tiab] OR "early retir*"[tiab] OR "effectiveness"[tiab] OR "employabil*"[tiab] OR "employment status*"[tiab] OR "job length*"[tiab] OR "job retenti*"[tiab] OR "job satisf*"[tiab] OR "work satisf*"[tiab] OR "medical certificate*"[tiab] OR "presenteeism*"[tiab] OR "productivit*"[tiab] OR "prolonged work*"[tiab] OR "prolonging work*"[tiab] OR "resignati*"[tiab] OR "return to work"[tiab] OR "sick leav*"[tiab] OR "sickness absen*"[tiab] OR "sickness presen*"[tiab] OR "sick listing*"[tiab] OR "sustainable work*"[tiab] OR "sustained work*"[tiab] OR "unemploy*"[tiab] OR "work absence*"[tiab] OR "work abilit*"[tiab] OR "work capacit*"[tiab] OR "work disabilit*"[tiab] OR "work engag*"[tiab] OR "work function*"[tiab] OR "work participati*"[tiab] OR "work performan*"[tiab] OR "work retention"[tiab] OR "turnover*"[tiab] OR "turn over*"[tiab])

**Cochrane**

([mh “Occupational Groups”] OR [mh "Occupational Health"] OR enterprise*:ti,ab OR business*:ti,ab OR "employed":ti,ab OR employee*:ti,ab OR employer*:ti,ab OR employment*:ti,ab OR (informal NEXT sector*):ti,ab OR (informal NEXT work*):ti,ab OR laborer*:ti,ab OR labourer*:ti,ab OR occupation*:ti,ab OR personnel*:ti,ab OR "professional":ti,ab OR "professionals":ti,ab OR "staff":ti,ab OR staffing*:ti,ab OR "vocation":ti,ab OR "vocations":ti,ab OR "worker":ti,ab OR "workers":ti,ab OR "workforce":ti,ab OR workplace*:ti,ab OR (work NEXT place*):ti,ab OR worksite*:ti,ab OR (work NEXT site*):ti,ab OR "aide":ti,ab OR "aides":ti,ab OR audiologist*:ti,ab OR ambulance*:ti,ab OR (care NEXT assistant*):ti,ab OR clinician*:ti,ab OR coastguard*:ti,ab OR (coast NEXT guard*):ti,ab OR dentist*:ti,ab OR detective*:ti,ab OR (disaster NEXT responder*):ti,ab OR doctor*:ti,ab OR "domestic health care":ti,ab OR (domiciliary NEXT care*):ti,ab OR (emergency NEXT service*):ti,ab OR (emergency NEXT responder*):ti,ab OR "emergency medical":ti,ab OR firefighter*:ti,ab OR (fire NEXT fighter*):ti,ab OR (first NEXT responder*):ti,ab OR (general NEXT practitioner*):ti,ab OR (health NEXT care NEXT provider*):ti,ab OR (healthcare NEXT provider*):ti,ab OR (health NEXT visitor*):ti,ab OR (home NEXT care NEXT service*):ti,ab OR (humanitarian NEXT aid*):ti,ab OR (humanitarian NEXT relie*):ti,ab OR (humanitarian NEXT service*):ti,ab OR (law NEXT enforc*):ti,ab OR lifeguard*:ti,ab OR (life NEXT guard*):ti,ab OR (medical NEXT resident*):ti,ab OR "medic":ti,ab OR "medics":ti,ab OR nurse*:ti,ab OR "nursing":ti,ab OR midwi*:ti,ab OR paramedic*:ti,ab OR "policemen":ti,ab OR "policeman":ti,ab OR "police men":ti,ab OR "police man":ti,ab OR "police women":ti,ab OR "police woman":ti,ab OR (police NEXT officer*):ti,ab OR "firemen":ti,ab OR "fireman":ti,ab OR "fire men":ti,ab OR "fire man":ti,ab OR "fire women":ti,ab OR "fire woman":ti,ab OR pharmacis*:ti,ab OR psychologist*:ti,ab OR physician*:ti,ab OR practitioner*:ti,ab OR (relief NEXT work*):ti,ab OR rescuer*:ti,ab OR (rescue NEXT work*):ti,ab OR therapist*:ti,ab OR veterinaria*:ti,ab)

AND

(((randomized NEXT trial*):ti,ab OR (randomised NEXT trial*):ti,ab OR (randomized NEXT control*):ti,ab OR (randomised NEXT control*):ti,ab OR (randomised NEXT design*):ti,ab OR (randomized NEXT design*):ti,ab) NOT (“letter”:pt OR “comment”:pt OR “case report” OR “editorial”:pt OR "Meta-Analysis":pt OR [mh "Meta-Analysis as Topic"] OR "Systematic Review":pt OR (([mh "Animals"] OR [mh "Models, Animal"]) NOT [mh humans])))

AND

((online*:ti,ab OR internet*:ti,ab OR web:ti,ab OR app:ti,ab OR apps:ti,ab OR computer*:ti,ab OR digital*:ti,ab OR smartphone*:ti,ab OR game*:ti,ab OR telemedicine*:ti,ab OR smart-phone*:ti,ab OR e-health:ti,ab OR ehealth:ti,ab OR mobile*:ti,ab OR mhealth:ti,ab OR m-health:ti,ab OR wearable*:ti,ab OR "smartwatch":ti,ab OR "smart-watch":ti,ab OR “biosensing”:ti,ab OR “wearable electronic device”:ti,ab OR “mobile app”:ti,ab OR "contactless":ti,ab OR electroencephalogra*:ti,ab OR "magnetic resonance imaging":ti,ab OR [mh "neurofeedback"] OR [mh "brain computer interfaces"] OR "fNIRS":ti,ab OR "alpha wave":ti,ab OR "EEG":ti,ab OR [mh “arrhythmias”] OR "cardiac output":ti,ab OR "photoplethysmography":ti,ab OR "PPG":ti,ab OR electrocardiogra*:ti,ab OR "ECG":ti,ab OR "RR interval":ti,ab OR "LF/HF":ti,ab OR "oxygen saturation":ti,ab OR "heart rate determination":ti,ab OR "rPPG":ti,ab OR "robotics":ti,ab OR "robot therapy":ti,ab OR "robot assisted therapy":ti,ab OR "chatbot":ti,ab OR "chatGPT":ti,ab OR “Virtual Reality”:ti,ab OR "VR":ti,ab OR "internet based intervention":ti,ab OR "metaverse":ti,ab OR "facial recognition":ti,ab OR "voice recognition":ti,ab OR "wavelet analysis":ti,ab OR "speech recognition software":ti,ab OR "phonetics":ti,ab OR "natural language processing":ti,ab OR "machine learning":ti,ab OR "artificial intelligence":ti,ab OR "deep learning":ti,ab OR "data mining":ti,ab OR “neural networks”:ti,ab OR “signal processing”:ti,ab OR "speech analysis":ti,ab OR "voice analysis":ti,ab OR "physiological signals":ti,ab OR "speech synthesis":ti,ab OR "motion capture":ti,ab OR "fitness trackers":ti,ab OR "accelerometry":ti,ab OR "actigraphy":ti,ab OR "biomechanical phenomena":ti,ab OR "ecological parameter monitoring":ti,ab OR electromyogra*:ti,ab OR “motion sensor”:ti,ab OR "gyroscope":ti,ab OR [mh "polysomnography"] OR [mh "sleep stages"] OR [mh "oximetry"] OR [mh "snoring"] OR electrooculogra*:ti,ab OR "heart rate variability":ti,ab OR "HRV":ti,ab OR "sleep efficiency":ti,ab OR "PSG":ti,ab OR "digital psychotherapy":ti,ab OR "thermometers":ti,ab OR "eye tracking":ti,ab OR "ultrasonics":ti,ab OR "photoacoustic techniques":ti,ab OR "photometry":ti,ab OR “text mining”:ti,ab OR “functional MRI”:ti,ab OR “fMRI”:ti,ab OR “heart rhythm”:ti,ab OR ”heart rate monitoring”:ti,ab OR “socially assistive robot”:ti,ab OR “facial expression recognition”:ti,ab OR “activity tracker”:ti,ab OR “biomechanics”:ti,ab OR “pulse oximeter”:ti,ab OR “SpO2”:ti,ab) OR ((online*:ti,ab OR internet*:ti,ab OR web:ti,ab OR app:ti,ab OR apps:ti,ab OR computer*:ti,ab OR digital*:ti,ab OR smartphone*:ti,ab OR game*:ti,ab OR telemedicine*:ti,ab OR smart-phone*:ti,ab OR e-health:ti,ab OR ehealth:ti,ab OR mobile*:ti,ab OR mhealth:ti,ab OR m-health:ti,ab OR wearable*:ti,ab OR "smartwatch":ti,ab OR "smart-watch":ti,ab OR “biosensing”:ti,ab OR “wearable electronic device”:ti,ab OR “mobile app”:ti,ab) AND ("heart rate":ti,ab OR "respiration":ti,ab OR "blood pressure":ti,ab OR "exercise":ti,ab OR "body temperature":ti,ab OR "sedentary behavior":ti,ab OR "walking":ti,ab OR "sleep":ti,ab OR "diet":ti,ab OR meal*:ti,ab OR "nutritional status":ti,ab OR "eating":ti,ab OR "energy intake":ti,ab OR "feeding behavior":ti,ab OR "food intake":ti,ab OR "crew resource management":ti,ab OR "mentoring":ti,ab OR "psychological safety":ti,ab OR "stress management training":ti,ab OR "teamwork training":ti,ab OR "communication training":ti,ab OR "cognitive behavioral therapy":ti,ab OR "play therapy":ti,ab OR "cognitive training":ti,ab OR "mindfulness":ti,ab OR "resistance":ti,ab OR “physical activity”:ti,ab OR "sedentary time":ti,ab OR “eating behavior”:ti,ab OR “music therapy”:ti,ab)))

AND

(([mh "Optimism"] OR [mh "Personal Satisfaction"] OR [mh "Self Concept"] OR [mh "Self Efficacy"] OR [mh "Self-Control"] OR (life NEXT engag*):ti,ab OR (life NEXT satisf*):ti,ab OR "meaning of life":ti,ab OR "purpose in life":ti,ab OR (positive NEXT affect*):ti,ab OR (positive NEXT emotion*):ti,ab OR resilien*:ti,ab OR (self NEXT concept*):ti,ab OR (self NEXT control*):ti,ab OR (self NEXT efficac*):ti,ab OR (self NEXT esteem*):ti,ab OR "swb":ti,ab OR (well NEXT being*):ti,ab OR wellbeing*:ti,ab) OR

([mh "Mental Disorders"] OR [mh "Mental Health"] OR [mh "Psychology, Industrial"] OR [mh "Stress, Psychological"] OR "adjustment":ti,ab OR (affective NEXT disorder*):ti,ab OR anxiet*:ti,ab OR bipolar*:ti,ab OR (burn NEXT out*):ti,ab OR burnout*:ti,ab OR "CMD":ti,ab OR depressi*:ti,ab OR (eating NEXT disorder*):ti,ab OR (mental NEXT disorder*):ti,ab OR (mental NEXT health*):ti,ab OR (mental NEXT illness*):ti,ab OR (mood NEXT disorder*):ti,ab OR (obsessive NEXT compulsive NEXT disorder*):ti,ab OR "ocd":ti,ab OR (panic NEXT disorder*):ti,ab OR phobi*:ti,ab OR (post NEXT traumatic*):ti,ab OR (psychiatric NEXT diagnos*):ti,ab OR (psychiatric NEXT disease*):ti,ab OR (psychiatric NEXT disorder*):ti,ab OR (psychiatric NEXT illness*):ti,ab OR (psychological NEXT disorder*):ti,ab OR psychos*:ti,ab OR psychotic*:ti,ab OR (psychological NEXT distress*):ti,ab OR "ptsd":ti,ab OR "ptss":ti,ab OR (somatoform NEXT disorder*):ti,ab OR schizophren*:ti,ab OR stress*:ti,ab) OR

([mh "Self-Injurious Behavior"] OR (auto NEXT mutilat*):ti,ab OR automutilat*:ti,ab OR (intentional NEXT injur*):ti,ab OR (self NEXT harm*):ti,ab OR selfharm*:ti,ab OR suicid*:ti,ab) OR

([mh "Substance-Related Disorders"] OR (alcohol NEXT abus*):ti,ab OR (alcohol NEXT misus*):ti,ab OR (alcohol NEXT us*):ti,ab OR alcoholis*:ti,ab OR amphetamin*:ti,ab OR (binge NEXT drinking*):ti,ab OR cannabis*:ti,ab OR cocain*:ti,ab OR diacetylmorphin*:ti,ab OR diamorphin*:ti,ab OR (drug NEXT abus*):ti,ab OR (drug NEXT misus*):ti,ab OR (drug NEXT us*):ti,ab OR drunk*:ti,ab OR "drinking":ti,ab OR ecstasy*:ti,ab OR "xtc":ti,ab OR fentanyl*:ti,ab OR hashish*:ti,ab OR heroin*:ti,ab OR marihuan*:ti,ab OR marijuan*:ti,ab OR "mdma":ti,ab OR methadon*:ti,ab OR methamphetamin*:ti,ab OR methylenedioxymethamphetamin*:ti,ab OR morphin*:ti,ab OR opiate*:ti,ab OR opioid*:ti,ab OR "thc":ti,ab OR (crystal NEXT meth*):ti,ab) OR

([mh "Quality of Life"] OR [mh "Quality-Adjusted Life Years"] OR "disability adjusted life":ti,ab OR "qaly":ti,ab OR "daly":ti,ab OR "functioning":ti,ab OR (functional NEXT abilit*):ti,ab OR functionalit*:ti,ab OR hrqol*:ti,ab OR (life NEXT activit*):ti,ab OR (life NEXT participati*):ti,ab OR (life NEXT stress*):ti,ab OR "qol":ti,ab OR "qoli":ti,ab OR (quality NEXT of NEXT life*):ti,ab OR (self NEXT car*):ti,ab OR selfcar*:ti,ab OR (sickness NEXT impact NEXT profile*):ti,ab OR (social NEXT function*):ti,ab OR (social NEXT participati*):ti,ab) OR

([mh "Absenteeism"] OR [mh "Employment"] OR [mh "Job Satisfaction"] OR [mh "Sick Leave"] OR [mh "Retirement"] OR [mh "Work Capacity Evaluation"] OR absenteeism*:ti,ab OR "back to work":ti,ab OR (early NEXT retir*):ti,ab OR "effectiveness":ti,ab OR employabil*:ti,ab OR (employment NEXT status*):ti,ab OR (job NEXT length*):ti,ab OR (job NEXT retenti*):ti,ab OR (job NEXT satisf*):ti,ab OR (work NEXT satisf*):ti,ab OR (medical NEXT certificate*):ti,ab OR presenteeism*:ti,ab OR productivit*:ti,ab OR (prolonged NEXT work*):ti,ab OR (prolonging NEXT work*):ti,ab OR resignati*:ti,ab OR "return to work":ti,ab OR (sick NEXT leav*):ti,ab OR (sickness NEXT absen*):ti,ab OR (sickness NEXT presen*):ti,ab OR (sick NEXT listing*):ti,ab OR (sustainable NEXT work*):ti,ab OR (sustained NEXT work*):ti,ab OR unemploy*:ti,ab OR (work NEXT absence*):ti,ab OR (work NEXT abilit*):ti,ab OR (work NEXT capacit*):ti,ab OR (work NEXT disabilit*):ti,ab OR (work NEXT engag*):ti,ab OR (work NEXT function*):ti,ab OR (work NEXT participati*):ti,ab OR (work NEXT performan*):ti,ab OR "work retention":ti,ab OR turnover*:ti,ab OR (turn NEXT over*):ti,ab))

**Embase**

(‘Occupational Groups’:de OR ‘Occupational Health’:de OR ‘enterprise*’:ti,ab OR ‘business*’:ti,ab OR ‘employed’:ti,ab OR ‘employee*’:ti,ab OR ‘employer*’:ti,ab OR ‘employment*’:ti,ab OR ‘informal sector*’:ti,ab OR ‘informal work*’:ti,ab OR ‘laborer*’:ti,ab OR ‘labourer*’:ti,ab OR ‘occupation*’:ti,ab OR ‘personnel*’:ti,ab OR ‘professional’:ti,ab OR ‘professionals’:ti,ab OR ‘staff’:ti,ab OR ‘staffing*’:ti,ab OR ‘vocation’:ti,ab OR ‘vocations’:ti,ab OR ‘worker’:ti,ab OR ‘workers’:ti,ab OR ‘workforce’:ti,ab OR ‘workplace*’:ti,ab OR ‘work place*’:ti,ab OR ‘worksite*’:ti,ab OR ‘work site*’:ti,ab OR ‘aide’:ti,ab OR ‘aides’:ti,ab OR ‘audiologist*’:ti,ab OR ‘ambulance*’:ti,ab OR ‘care assistant*’:ti,ab OR ‘clinician*’:ti,ab OR ‘coastguard*’:ti,ab OR ‘coast guard*’:ti,ab OR ‘dentist*’:ti,ab OR ‘detective*’:ti,ab OR ‘disaster responder*’:ti,ab OR ‘doctor*’:ti,ab OR ‘domestic health care’:ti,ab OR ‘domiciliary care*’:ti,ab OR ‘emergency service*’:ti,ab OR ‘emergency responder*’:ti,ab OR ‘emergency medical’:ti,ab OR ‘firefighter*’:ti,ab OR ‘fire fighter*’:ti,ab OR ‘first responder*’:ti,ab OR ‘general practitioner*’:ti,ab OR ‘health care provider*’:ti,ab OR ‘healthcare provider*’:ti,ab OR ‘health visitor*’:ti,ab OR ‘home care service*’:ti,ab OR ‘humanitarian aid*’:ti,ab OR ‘humanitarian relie*’:ti,ab OR ‘humanitarian service*’:ti,ab OR ‘law enforc*’:ti,ab OR ‘lifeguard*’:ti,ab OR ‘life guard*’:ti,ab OR ‘medical resident*’:ti,ab OR ‘medic’:ti,ab OR ‘medics’:ti,ab OR ‘nurse*’:ti,ab OR ‘nursing’:ti,ab OR ‘midwi*’:ti,ab OR ‘paramedic*’:ti,ab OR ‘policemen’:ti,ab OR ‘policeman’:ti,ab OR ‘police men’:ti,ab OR ‘police man’:ti,ab OR ‘police women’:ti,ab OR ‘police woman’:ti,ab OR ‘police officer*’:ti,ab OR ‘firemen’:ti,ab OR ‘fireman’:ti,ab OR ‘fire men’:ti,ab OR ‘fire man’:ti,ab OR ‘fire women’:ti,ab OR ‘fire woman’:ti,ab OR ‘pharmacis*’:ti,ab OR ‘psychologist*’:ti,ab OR ‘physician*’:ti,ab OR ‘practitioner*’:ti,ab OR ‘relief work*’:ti,ab OR ‘rescuer*’:ti,ab OR ‘rescue work*’:ti,ab OR ‘therapist*’:ti,ab OR ‘veterinaria*’:ti,ab)

AND

(((randomized:ti,ab AND trial*:ti,ab) OR (randomised:ti,ab AND trial*:ti,ab) OR ‘randomized control*’:ti,ab OR ‘randomised control*’:ti,ab OR ‘randomised design*’:ti,ab OR ‘randomized design*’:ti,ab) NOT (‘letter’:it OR ‘comment’:it OR ‘case report’ OR ‘editorial’:it OR ‘Meta-Analysis’:it OR ‘Systematic Review’:it OR ((‘Animals’:de OR ‘Models, Animal’:de) NOT humans:de)))

AND

((online*:ti,ab OR internet*:ti,ab OR web:ti,ab OR app:ti,ab OR apps:ti,ab OR computer*:ti,ab OR digital*:ti,ab OR smartphone*:ti,ab OR game*:ti,ab OR telemedicine*:ti,ab OR smart-phone*:ti,ab OR e-health:ti,ab OR ehealth:ti,ab OR mobile*:ti,ab OR mhealth:ti,ab OR m-health:ti,ab OR wearable*:ti,ab OR ‘smartwatch’:ti,ab OR ‘smart-watch’:ti,ab OR ‘biosensing’:ti,ab OR ‘wearable electronic device’:ti,ab OR ‘mobile app’:ti,ab OR ‘contactless’:ti,ab OR electroencephalogra*:ti,ab OR ‘magnetic resonance imaging’:ti,ab OR ‘neurofeedback’:de OR ‘brain computer interfaces’:de OR ‘fNIRS’:ti,ab OR ‘alpha wave’:ti,ab OR ‘EEG’:ti,ab OR arrhythmias:de OR ‘cardiac output’:ti,ab OR ‘photoplethysmography’:ti,ab OR ‘PPG’:ti,ab OR electrocardiogra*:ti,ab OR ‘ECG’:ti,ab OR ‘RR interval’:ti,ab OR ‘LF/HF’:ti,ab OR ‘oxygen saturation’:ti,ab OR ‘heart rate determination’:ti,ab OR ‘rPPG’:ti,ab OR ‘robotics’:ti,ab OR ‘robot therapy’:ti,ab OR ‘robot assisted therapy’:ti,ab OR ‘chatbot’:ti,ab OR ‘chatGPT’:ti,ab OR ‘Virtual Reality’:ti,ab OR ‘VR’:ti,ab OR ‘internet based intervention’:ti,ab OR ‘metaverse’:ti,ab OR ‘facial recognition’:ti,ab OR ‘voice recognition’:ti,ab OR ‘wavelet analysis’:ti,ab OR ‘speech recognition software’:ti,ab OR ‘phonetics’:ti,ab OR ‘natural language processing’:ti,ab OR ‘machine learning’:ti,ab OR ‘artificial intelligence’:ti,ab OR ‘deep learning’:ti,ab OR ‘data mining’:ti,ab OR ‘neural networks’:ti,ab OR ‘signal processing’:ti,ab OR ‘speech analysis’:ti,ab OR ‘voice analysis’:ti,ab OR ‘physiological signals’:ti,ab OR ‘speech synthesis’:ti,ab OR ‘motion capture’:ti,ab OR ‘fitness trackers’:ti,ab OR ‘accelerometry’:ti,ab OR ‘actigraphy’:ti,ab OR ‘biomechanical phenomena’:ti,ab OR ‘ecological parameter monitoring’:ti,ab OR electromyogra*:ti,ab OR ‘motion sensor’:ti,ab OR ‘gyroscope’:ti,ab OR ‘polysomnography’:de OR ‘sleep stages’:de OR ‘oximetry’:de OR ‘snoring’:de OR electrooculogra*:ti,ab OR ‘heart rate variability’:ti,ab OR ‘HRV’:ti,ab OR ‘sleep efficiency’:ti,ab OR ‘PSG’:ti,ab OR ‘digital psychotherapy’:ti,ab OR ‘thermometers’:ti,ab OR ‘eye tracking’:ti,ab OR ‘ultrasonics’:ti,ab OR ‘photoacoustic techniques’:ti,ab OR ‘photometry’:ti,ab OR ‘text mining’:ti,ab OR ‘functional MRI’:ti,ab OR ‘fMRI’:ti,ab OR ‘heart rhythm’:ti,ab OR ‘heart rate monitoring’:ti,ab OR ‘socially assistive robot’:ti,ab OR ‘facial expression recognition’:ti,ab OR ‘activity tracker’:ti,ab OR ‘biomechanics’:ti,ab OR ‘pulse oximeter’:ti,ab OR ‘SpO2’:ti,ab) OR ((online*:ti,ab OR internet*:ti,ab OR web:ti,ab OR app:ti,ab OR apps:ti,ab OR computer*:ti,ab OR digital*:ti,ab OR smartphone*:ti,ab OR game*:ti,ab OR telemedicine*:ti,ab OR smart-phone*:ti,ab OR e-health:ti,ab OR ehealth:ti,ab OR mobile*:ti,ab OR mhealth:ti,ab OR m-health:ti,ab OR wearable*:ti,ab OR ‘smartwatch’:ti,ab OR ‘smart-watch’:ti,ab OR ‘biosensing’:ti,ab OR ‘wearable electronic device’:ti,ab OR ‘mobile app’:ti,ab) AND (‘heart rate’:ti,ab OR ‘respiration’:ti,ab OR ‘blood pressure’:ti,ab OR ‘exercise’:ti,ab OR ‘body temperature’:ti,ab OR ‘sedentary behavior’:ti,ab OR ‘walking’:ti,ab OR ‘sleep’:ti,ab OR ‘diet’:ti,ab OR meal*:ti,ab OR ‘nutritional status’:ti,ab OR ‘eating’:ti,ab OR ‘energy intake’:ti,ab OR ‘feeding behavior’:ti,ab OR ‘food intake’:ti,ab OR ‘crew resource management’:ti,ab OR ‘mentoring’:ti,ab OR ‘psychological safety’:ti,ab OR ‘stress management training’:ti,ab OR ‘teamwork training’:ti,ab OR ‘communication training’:ti,ab OR ‘cognitive behavioral therapy’:ti,ab OR ‘play therapy’:ti,ab OR ‘cognitive training’:ti,ab OR ‘mindfulness’:ti,ab OR ‘resistance’:ti,ab OR ‘physical activity’:ti,ab OR ‘sedentary time’:ti,ab OR ‘eating behavior’:ti,ab OR ‘music therapy’:ti,ab)))

AND

((‘Optimism’:de OR ‘Personal Satisfaction’:de OR ‘Self Concept’:de OR ‘Self Efficacy’:de OR ‘Self-Control’:de OR ‘life engag*’:ti,ab OR ‘life satisf*’:ti,ab OR ‘meaning of life’:ti,ab OR ‘purpose in life’:ti,ab OR ‘positive affect*’:ti,ab OR ‘positive emotion*’:ti,ab OR ‘resilien*’:ti,ab OR ‘self concept*’:ti,ab OR ‘self control*’:ti,ab OR ‘self efficac*’:ti,ab OR ‘self esteem*’:ti,ab OR ‘swb’:ti,ab OR ‘well being*’:ti,ab OR ‘wellbeing*’:ti,ab) OR

(‘Mental Disorders’:de OR ‘Mental Health’:de OR ‘Psychology, Industrial’:de OR ‘Stress, Psychological’:de OR ‘adjustment’:ti,ab OR ‘affective disorder*’:ti,ab OR ‘anxiet*’:ti,ab OR ‘bipolar*’:ti,ab OR ‘burn out*’:ti,ab OR ‘burnout*’:ti,ab OR ‘CMD’:ti,ab OR ‘depressi*’:ti,ab OR ‘eating disorder*’:ti,ab OR ‘mental disorder*’:ti,ab OR ‘mental health*’:ti,ab OR ‘mental illness*’:ti,ab OR ‘mood disorder*’:ti,ab OR ‘obsessive compulsive disorder*’:ti,ab OR ‘ocd’:ti,ab OR ‘panic disorder*’:ti,ab OR ‘phobi*’:ti,ab OR ‘post traumatic*’:ti,ab OR ‘psychiatric diagnos*’:ti,ab OR ‘psychiatric disease*’:ti,ab OR ‘psychiatric disorder*’:ti,ab OR ‘psychiatric illness*’:ti,ab OR ‘psychological disorder*’:ti,ab OR ‘psychos*’:ti,ab OR ‘psychotic*’:ti,ab OR ‘psychological distress*’:ti,ab OR ‘ptsd’:ti,ab OR ‘ptss’:ti,ab OR ‘somatoform disorder*’:ti,ab OR ‘schizophren*’:ti,ab OR ‘stress*’:ti,ab) OR

(‘Self-Injurious Behavior’:de OR ‘auto mutilat*’:ti,ab OR ‘automutilat*’:ti,ab OR ‘intentional injur*’:ti,ab OR ‘self harm*’:ti,ab OR ‘selfharm*’:ti,ab OR ‘suicid*’:ti,ab) OR

(‘Substance-Related Disorders’:de OR ‘alcohol abus*’:ti,ab OR ‘alcohol misus*’:ti,ab OR ‘alcohol us*’:ti,ab OR ‘alcoholis*’:ti,ab OR ‘amphetamin*’:ti,ab OR ‘binge drinking*’:ti,ab OR ‘cannabis*’:ti,ab OR ‘cocain*’:ti,ab OR ‘diacetylmorphin*’:ti,ab OR ‘diamorphin*’:ti,ab OR ‘drug abus*’:ti,ab OR ‘drug misus*’:ti,ab OR ‘drug us*’:ti,ab OR ‘drunk*’:ti,ab OR ‘drinking’:ti,ab OR ‘ecstasy*’:ti,ab OR ‘xtc’:ti,ab OR ‘fentanyl*’:ti,ab OR ‘hashish*’:ti,ab OR ‘heroin*’:ti,ab OR ‘marihuan*’:ti,ab OR ‘marijuan*’:ti,ab OR ‘mdma’:ti,ab OR ‘methadon*’:ti,ab OR ‘methamphetamin*’:ti,ab OR ‘methylenedioxymethamphetamin*’:ti,ab OR ‘morphin*’:ti,ab OR ‘opiate*’:ti,ab OR ‘opioid*’:ti,ab OR ‘thc’:ti,ab OR ‘crystal meth*’:ti,ab) OR

(‘Quality of Life’:de OR ‘Quality-Adjusted Life Years’:de OR ‘disability adjusted life’:ti,ab OR ‘qaly’:ti,ab OR ‘daly’:ti,ab OR ‘functioning’:ti,ab OR ‘functional abilit*’:ti,ab OR ‘functionalit*’:ti,ab OR ‘hrqol*’:ti,ab OR ‘life activit*’:ti,ab OR ‘life participati*’:ti,ab OR ‘life stress*’:ti,ab OR ‘qol’:ti,ab OR ‘qoli’:ti,ab OR ‘quality of life*’:ti,ab OR ‘self car*’:ti,ab OR ‘selfcar*’:ti,ab OR ‘sickness impact profile*’:ti,ab OR ‘social function*’:ti,ab OR ‘social participati*’:ti,ab) OR

(‘Absenteeism’:de OR ‘Employment’:de OR ‘Job Satisfaction’:de OR ‘Sick Leave’:de OR ‘Retirement’:de OR ‘Work Capacity Evaluation’:de OR ‘absenteeism*’:ti,ab OR ‘back to work’:ti,ab OR ‘early retir*’:ti,ab OR ‘effectiveness’:ti,ab OR ‘employabil*’:ti,ab OR ‘employment status*’:ti,ab OR ‘job length*’:ti,ab OR ‘job retenti*’:ti,ab OR ‘job satisf*’:ti,ab OR ‘work satisf*’:ti,ab OR ‘medical certificate*’:ti,ab OR ‘presenteeism*’:ti,ab OR ‘productivit*’:ti,ab OR ‘prolonged work*’:ti,ab OR ‘prolonging work*’:ti,ab OR ‘resignati*’:ti,ab OR ‘return to work’:ti,ab OR ‘sick leav*’:ti,ab OR ‘sickness absen*’:ti,ab OR ‘sickness presen*’:ti,ab OR ‘sick listing*’:ti,ab OR ‘sustainable work*’:ti,ab OR ‘sustained work*’:ti,ab OR ‘unemploy*’:ti,ab OR ‘work absence*’:ti,ab OR ‘work abilit*’:ti,ab OR ‘work capacit*’:ti,ab OR ‘work disabilit*’:ti,ab OR ‘work engag*’:ti,ab OR ‘work function*’:ti,ab OR ‘work participati*’:ti,ab OR ‘work performan*’:ti,ab OR ‘work retention’:ti,ab OR ‘turnover*’:ti,ab OR ‘turn over*’:ti,ab))

**PsycINFO/ARTICLES**

(MA "Occupational Groups" OR MA "Occupational Health" OR TI “enterprise*” OR TI "business*" OR TI "employed" OR TI "employee*" OR TI "employer*" OR TI "employment*" OR TI "informal sector*" OR TI "informal work*" OR TI "laborer*" OR TI "labourer*" OR TI "occupation*" OR TI "personnel*" OR TI "professional" OR TI "professionals" OR TI "staff" OR TI "staffing*" OR TI "vocation" OR TI "vocations" OR TI "worker" OR TI "workers" OR TI "workforce" OR TI "workplace*" OR TI "work place*" OR TI "worksite*" OR TI "work site*" OR TI "aide" OR TI "aides" OR TI "audiologist*" OR TI "ambulance*" OR TI "care assistant*" OR TI "clinician*" OR TI "coastguard*" OR TI "coast guard*" OR TI "dentist*" OR TI "detective*" OR TI "disaster responder*" OR TI "doctor*" OR TI "domestic health care" OR TI "domiciliary care*" OR TI "emergency service*" OR TI "emergency responder*" OR TI "emergency medical" OR TI "firefighter*" OR TI "fire fighter*" OR TI "first responder*" OR TI "general practitioner*" OR TI "health care provider*" OR TI "healthcare provider*" OR TI "health visitor*" OR TI "home care service*" OR TI "humanitarian aid*" OR TI "humanitarian relie*" OR TI "humanitarian service*" OR TI "law enforc*" OR TI "lifeguard*" OR TI "life guard*" OR TI "medical resident*" OR TI "medic" OR TI "medics" OR TI "nurse*" OR TI "nursing" OR TI "midwi*" OR TI "paramedic*" OR TI "policemen" OR TI "policeman" OR TI "police men" OR TI "police man" OR TI "police women" OR TI "police woman" OR TI "police officer*" OR TI "firemen" OR TI "fireman" OR TI "fire men" OR TI "fire man" OR TI "fire women" OR TI "fire woman" OR TI "pharmacis*" OR TI "psychologist*" OR TI "physician*" OR TI "practitioner*" OR TI "relief work*" OR TI "rescuer*" OR TI "rescue work*" OR TI "therapist*" OR TI "veterinaria*" OR AB “enterprise*” OR AB "business*" OR AB "employed" OR AB "employee*" OR AB "employer*" OR AB "employment*" OR AB "informal sector*" OR AB "informal work*" OR AB "laborer*" OR AB "labourer*" OR AB "occupation*" OR AB "personnel*" OR AB "professional" OR AB "professionals" OR AB "staff" OR AB "staffing*" OR AB "vocation" OR AB "vocations" OR AB "worker" OR AB "workers" OR AB "workforce" OR AB "workplace*" OR AB "work place*" OR AB "worksite*" OR AB "work site*" OR AB "aide" OR AB "aides" OR AB "audiologist*" OR AB "ambulance*" OR AB "care assistant*" OR AB "clinician*" OR AB "coastguard*" OR AB "coast guard*" OR AB "dentist*" OR AB "detective*" OR AB "disaster responder*" OR AB "doctor*" OR AB "domestic health care" OR AB "domiciliary care*" OR AB "emergency service*" OR AB "emergency responder*" OR AB "emergency medical" OR AB "firefighter*" OR AB "fire fighter*" OR AB "first responder*" OR AB "general practitioner*" OR AB "health care provider*" OR AB "healthcare provider*" OR AB "health visitor*" OR AB "home care service*" OR AB "humanitarian aid*" OR AB "humanitarian relie*" OR AB "humanitarian service*" OR AB "law enforc*" OR AB "lifeguard*" OR AB "life guard*" OR AB "medical resident*" OR AB "medic" OR AB "medics" OR AB "nurse*" OR AB "nursing" OR AB "midwi*" OR AB "paramedic*" OR AB "policemen" OR AB "policeman" OR AB "police men" OR AB "police man" OR AB "police women" OR AB "police woman" OR AB "police officer*" OR AB "firemen" OR AB "fireman" OR AB "fire men" OR AB "fire man" OR AB "fire women" OR AB "fire woman" OR AB "pharmacis*" OR AB "psychologist*" OR AB "physician*" OR AB "practitioner*" OR AB "relief work*" OR AB "rescuer*" OR AB "rescue work*" OR AB "therapist*" OR AB "veterinaria*" )

AND

(((TI randomized AND TI trial*) OR (TI randomised AND TI trial*) OR TI "randomized control*" OR TI "randomised control*" OR TI "randomised design*" OR TI "randomized design*" OR (AB randomized AND AB trial*) OR (AB randomised AND AB trial*) OR AB "randomized control*" OR AB "randomised control*" OR AB "randomised design*" OR AB "randomized design*") NOT (PT "Comment" OR PT "Letter" OR TX case report OR PT "Editorial" OR PT "Meta-Analysis" OR MA "Meta-Analysis as Topic" OR PT "Systematic Review" OR ((MA "Animals" OR MA "Models, Animal") NOT MA "Humans")))

AND

((TI online* OR TI internet* OR TI web OR TI app OR TI apps OR TI computer* OR TI digital* OR TI smartphone* OR TI game* OR TI telemedicine* OR TI smart-phone* OR TI e-health OR TI ehealth OR TI mobile* OR TI mhealth OR TI m-health OR TI wearable* OR TI "smartwatch" OR TI "smart-watch" OR TI “biosensing” OR TI “wearable electronic device” OR TI “mobile app” OR TI "contactless" OR TI electroencephalogra* OR TI "magnetic resonance imaging" OR TI "neurofeedback"[Mesh] OR MA "brain computer interfaces" OR TI "fNIRS" OR TI "alpha wave" OR TI "EEG" OR MA arrhythmias OR TI "cardiac output" OR TI "photoplethysmography" OR TI "PPG" OR TI electrocardiogra* OR TI "ECG" OR TI "RR interval" OR TI "LF/HF" OR TI "oxygen saturation" OR TI "heart rate determination" OR TI "rPPG" OR TI "robotics" OR TI "robot therapy" OR TI "robot assisted therapy" OR TI "chatbot" OR TI "chatGPT" OR TI “Virtual Reality” OR TI "VR" OR TI "internet based intervention" OR TI "metaverse" OR TI "facial recognition" OR TI "voice recognition" OR TI "wavelet analysis" OR TI "speech recognition software" OR TI "phonetics" OR TI "natural language processing" OR TI "machine learning" OR TI "artificial intelligence" OR TI "deep learning" OR TI "data mining" OR TI “neural networks” OR TI “signal processing” OR TI "speech analysis" OR TI "voice analysis" OR TI "physiological signals" OR TI "speech synthesis" OR TI "motion capture" OR TI "fitness trackers" OR TI "accelerometry" OR TI "actigraphy" OR TI "biomechanical phenomena" OR TI "ecological parameter monitoring" OR TI electromyogra* OR TI “motion sensor” OR TI "gyroscope" OR MA "polysomnography" OR MA "sleep stages" OR MA "oximetry" OR MA "snoring" OR TI electrooculogra* OR TI "heart rate variability" OR TI "HRV" OR TI "sleep efficiency" OR TI "PSG" OR TI "digital psychotherapy" OR TI "thermometers" OR TI "eye tracking" OR TI "ultrasonics" OR TI "photoacoustic techniques" OR TI "photometry" OR TI “text mining” OR TI “functional MRI” OR TI “fMRI” OR TI “heart rhythm” OR TI ”heart rate monitoring” OR TI “socially assistive robot” OR TI “facial expression recognition” OR TI “activity tracker” OR TI “biomechanics” OR TI “pulse oximeter” OR TI “SpO2”) OR ((TI online* OR TI internet* OR TI web OR TI app OR TI apps OR TI computer* OR TI digital* OR TI smartphone* OR TI game* OR TI telemedicine* OR TI smart-phone* OR TI e-health OR TI ehealth OR TI mobile* OR TI mhealth OR TI m-health OR TI wearable* OR TI "smartwatch" OR TI "smart-watch" OR TI “biosensing” OR TI “wearable electronic device” OR TI “mobile app” ) AND (TI "heart rate" OR TI "respiration" OR TI "blood pressure" OR TI "exercise" OR TI "body temperature" OR TI "sedentary behavior" OR TI "walking" OR TI "sleep" OR TI "diet" OR TI meal* OR TI "nutritional status" OR TI "eating" OR TI "energy intake" OR TI "feeding behavior" OR TI "food intake" OR TI "crew resource management" OR TI "mentoring" OR TI "psychological safety" OR TI "stress management training" OR TI "teamwork training" OR TI "communication training" OR TI "cognitive behavioral therapy" OR TI "play therapy" OR TI "cognitive training" OR TI "mindfulness" OR TI "resistance" OR TI “physical activity” OR TI "sedentary time" OR TI “eating behavior” OR TI “music therapy”)) OR

(AB online* OR AB internet* OR AB web OR AB app OR AB apps OR AB computer* OR AB digital* OR AB smartphone* OR AB game* OR AB telemedicine* OR AB smart-phone* OR AB e-health OR AB ehealth OR AB mobile* OR AB mhealth OR AB m-health OR AB wearable* OR AB "smartwatch" OR AB "smart-watch" OR AB “biosensing” OR AB “wearable electronic device” OR AB “mobile app” OR AB "contactless" OR AB electroencephalogra* OR AB "magnetic resonance imaging" OR AB "neurofeedback"[Mesh] OR MA "brain computer interfaces" OR AB "fNIRS" OR AB "alpha wave" OR AB "EEG" OR MA arrhythmias OR AB "cardiac output" OR AB "photoplethysmography" OR AB "PPG" OR AB electrocardiogra* OR AB "ECG" OR AB "RR interval" OR AB "LF/HF" OR AB "oxygen saturation" OR AB "heart rate determination" OR AB "rPPG" OR AB "robotics" OR AB "robot therapy" OR AB "robot assisted therapy" OR AB "chatbot" OR AB "chatGPT" OR AB “Virtual Reality” OR AB "VR" OR AB "internet based intervention" OR AB "metaverse" OR AB "facial recognition" OR AB "voice recognition" OR AB "wavelet analysis" OR AB "speech recognition software" OR AB "phonetics" OR AB "natural language processing" OR AB "machine learning" OR AB "artificial intelligence" OR AB "deep learning" OR AB "data mining" OR AB “neural networks” OR AB “signal processing” OR AB "speech analysis" OR AB "voice analysis" OR AB "physiological signals" OR AB "speech synthesis" OR AB "motion capture" OR AB "fitness trackers" OR AB "accelerometry" OR AB "actigraphy" OR AB "biomechanical phenomena" OR AB "ecological parameter monitoring" OR AB electromyogra* OR AB “motion sensor” OR AB "gyroscope" OR MA "polysomnography" OR MA "sleep stages" OR MA "oximetry" OR MA "snoring" OR AB electrooculogra* OR AB "heart rate variability" OR AB "HRV" OR AB "sleep efficiency" OR AB "PSG" OR AB "digital psychotherapy" OR AB "thermometers" OR AB "eye tracking" OR AB "ultrasonics" OR AB "photoacoustic techniques" OR AB "photometry" OR AB “text mining” OR AB “functional MRI” OR AB “fMRI” OR AB “heart rhythm” OR AB ”heart rate monitoring” OR AB “socially assistive robot” OR AB “facial expression recognition” OR AB “activity tracker” OR AB “biomechanics” OR AB “pulse oximeter” OR AB “SpO2”) OR ((AB online* OR AB internet* OR AB web OR AB app OR AB apps OR AB computer* OR AB digital* OR AB smartphone* OR AB game* OR AB telemedicine* OR AB smart-phone* OR AB e-health OR AB ehealth OR AB mobile* OR AB mhealth OR AB m-health OR AB wearable* OR AB "smartwatch" OR AB "smart-watch" OR AB “biosensing” OR AB “wearable electronic device” OR AB “mobile app” ) AND (AB "heart rate" OR AB "respiration" OR AB "blood pressure" OR AB "exercise" OR AB "body temperature" OR AB "sedentary behavior" OR AB "walking" OR AB "sleep" OR AB "diet" OR AB meal* OR AB "nutritional status" OR AB "eating" OR AB "energy intake" OR AB "feeding behavior" OR AB "food intake" OR AB "crew resource management" OR AB "mentoring" OR AB "psychological safety" OR AB "stress management training" OR AB "teamwork training" OR AB "communication training" OR AB "cognitive behavioral therapy" OR AB "play therapy" OR AB "cognitive training" OR AB "mindfulness" OR AB "resistance" OR AB “physical activity” OR AB "sedentary time" OR AB “eating behavior” OR AB “music therapy”)))

AND

((MA "Optimism" OR MA "Personal Satisfaction" OR MA "Self Concept" OR MA "Self Efficacy" OR MA "Self-Control" OR TI "life engag*" OR TI "life satisf*" OR TI "meaning of life" OR TI "purpose in life" OR TI "positive affect*" OR TI "positive emotion*" OR TI "resilien*" OR TI "self concept*" OR TI "self control*" OR TI "self efficac*" OR TI "self esteem*" OR TI "swb" OR TI "well being*" OR TI "wellbeing*" OR AB "life engag*" OR AB "life satisf*" OR AB "meaning of life" OR AB "purpose in life" OR AB "positive affect*" OR AB "positive emotion*" OR AB "resilien*" OR AB "self concept*" OR AB "self control*" OR AB "self efficac*" OR AB "self esteem*" OR AB "swb" OR AB "well being*" OR AB "wellbeing*" ) OR

(MA "Mental Disorders" OR MA "Mental Health" OR MA "Psychology, Industrial" OR MA "Stress, Psychological" OR TI "adjustment" OR TI "affective disorder*" OR TI "anxiet*" OR TI "bipolar*" OR TI "burn out*" OR TI "burnout*" OR TI "CMD" OR TI "depressi*" OR TI "eating disorder*" OR TI "mental disorder*" OR TI "mental health*" OR TI "mental illness*" OR TI "mood disorder*" OR TI "obsessive compulsive disorder*" OR TI "ocd" OR TI "panic disorder*" OR TI "phobi*" OR TI "post traumatic*" OR TI "psychiatric diagnos*" OR TI "psychiatric disease*" OR TI "psychiatric disorder*" OR TI "psychiatric illness*" OR TI "psychological disorder*" OR TI "psychos*" OR TI "psychotic*" OR TI "psychological distress*" OR TI "ptsd" OR TI "ptss" OR TI "somatoform disorder*" OR TI "schizophren*" OR TI "stress*" OR AB "adjustment" OR AB "affective disorder*" OR AB "anxiet*" OR AB "bipolar*" OR AB "burn out*" OR AB "burnout*" OR AB "CMD" OR AB "depressi*" OR AB "eating disorder*" OR AB "mental disorder*" OR AB "mental health*" OR AB "mental illness*" OR AB "mood disorder*" OR AB "obsessive compulsive disorder*" OR AB "ocd" OR AB "panic disorder*" OR AB "phobi*" OR AB "post traumatic*" OR AB "psychiatric diagnos*" OR AB "psychiatric disease*" OR AB "psychiatric disorder*" OR AB "psychiatric illness*" OR AB "psychological disorder*" OR AB "psychos*" OR AB "psychotic*" OR AB "psychological distress*" OR AB "ptsd" OR AB "ptss" OR AB "somatoform disorder*" OR AB "schizophren*" OR AB "stress*" ) OR

(MA "Self-Injurious Behavior" OR TI "auto mutilat*" OR TI "automutilat*" OR TI "intentional injur*" OR TI "self harm*" OR TI "selfharm*" OR TI "suicid*" OR AB "auto mutilat*" OR AB "automutilat*" OR AB "intentional injur*" OR AB "self harm*" OR AB "selfharm*" OR AB "suicid*" ) OR

(MA "Substance-Related Disorders" OR TI "alcohol abus*" OR TI "alcohol misus*" OR TI "alcohol us*" OR TI "alcoholis*" OR TI "amphetamin*" OR TI "binge drinking*" OR TI "cannabis*" OR TI "cocain*" OR TI "diacetylmorphin*" OR TI "diamorphin*" OR TI "drug abus*" OR TI "drug misus*" OR TI "drug us*" OR TI "drunk*" OR TI "drinking" OR TI "ecstasy*" OR TI "xtc" OR TI "fentanyl*" OR TI "hashish*" OR TI "heroin*" OR TI "marihuan*" OR TI "marijuan*" OR TI "mdma" OR TI "methadon*" OR TI "methamphetamin*" OR TI "methylenedioxymethamphetamin*" OR TI "morphin*" OR TI "opiate*" OR TI "opioid*" OR TI "thc" OR TI "crystal meth*" OR AB "alcohol abus*" OR AB "alcohol misus*" OR AB "alcohol us*" OR AB "alcoholis*" OR AB "amphetamin*" OR AB "binge drinking*" OR AB "cannabis*" OR AB "cocain*" OR AB "diacetylmorphin*" OR AB "diamorphin*" OR AB "drug abus*" OR AB "drug misus*" OR AB "drug us*" OR AB "drunk*" OR AB "drinking" OR AB "ecstasy*" OR AB "xtc" OR AB "fentanyl*" OR AB "hashish*" OR AB "heroin*" OR AB "marihuan*" OR AB "marijuan*" OR AB "mdma" OR AB "methadon*" OR AB "methamphetamin*" OR AB "methylenedioxymethamphetamin*" OR AB "morphin*" OR AB "opiate*" OR AB "opioid*" OR AB "thc" OR AB "crystal meth*" ) OR

(MA "Quality of Life" OR MA "Quality-Adjusted Life Years" OR TI "disability adjusted life" OR TI "qaly" OR TI "daly" OR TI "functioning" OR TI "functional abilit*" OR TI "functionalit*" OR TI "hrqol*" OR TI "life activit*" OR TI "life participati*" OR TI "life stress*" OR TI "qol" OR TI "qoli" OR TI "quality of life*" OR TI "self car*" OR TI "selfcar*" OR TI "sickness impact profile*" OR TI "social function*" OR TI "social participati*" OR AB "disability adjusted life" OR AB "qaly" OR AB "daly" OR AB "functioning" OR AB "functional abilit*" OR AB "functionalit*" OR AB "hrqol*" OR AB "life activit*" OR AB "life participati*" OR AB "life stress*" OR AB "qol" OR AB "qoli" OR AB "quality of life*" OR AB "self car*" OR AB "selfcar*" OR AB "sickness impact profile*" OR AB "social function*" OR AB "social participati*" ) OR

(MA "Absenteeism" OR MA "Employment" OR MA "Job Satisfaction" OR MA "Sick Leave" OR MA "Retirement" OR MA "Work Capacity Evaluation" OR TI "absenteeism*" OR TI "back to work" OR TI "early retir*" OR TI "effectiveness" OR TI "employabil*" OR TI "employment status*" OR TI "job length*" OR TI "job retenti*" OR TI "job satisf*" OR TI "work satisf*" OR TI "medical certificate*" OR TI "presenteeism*" OR TI "productivit*" OR TI "prolonged work*" OR TI "prolonging work*" OR TI "resignati*" OR TI "return to work" OR TI "sick leav*" OR TI "sickness absen*" OR TI "sickness presen*" OR TI "sick listing*" OR TI "sustainable work*" OR TI "sustained work*" OR TI "unemploy*" OR TI "work absence*" OR TI "work abilit*" OR TI "work capacit*" OR TI "work disabilit*" OR TI "work engag*" OR TI "work function*" OR TI "work participati*" OR TI "work performan*" OR TI "work retention" OR TI "turnover*" OR TI "turn over*" OR AB "absenteeism*" OR AB "back to work" OR AB "early retir*" OR AB "effectiveness" OR AB "employabil*" OR AB "employment status*" OR AB "job length*" OR AB "job retenti*" OR AB "job satisf*" OR AB "work satisf*" OR AB "medical certificate*" OR AB "presenteeism*" OR AB "productivit*" OR AB "prolonged work*" OR AB "prolonging work*" OR AB "resignati*" OR AB "return to work" OR AB "sick leav*" OR AB "sickness absen*" OR AB "sickness presen*" OR AB "sick listing*" OR AB "sustainable work*" OR AB "sustained work*" OR AB "unemploy*" OR AB "work absence*" OR AB "work abilit*" OR AB "work capacit*" OR AB "work disabilit*" OR AB "work engag*" OR AB "work function*" OR AB "work participati*" OR AB "work performan*" OR AB "work retention" OR AB "turnover*" OR AB "turn over*" ))

**Japan Medical Abstract Society database**

((職業別集団/TH or 職業別集団/AL) or (労働衛生/TH or 労働衛生/AL) or 企業/AL or (商業/TH or ビジネス/AL) or (雇用/TH or 雇用/AL) or 従業員/AL or 雇用主/AL or (インフォーマルセクター/TH or 非公式セクター/AL) or 非公式な仕事/AL or (労働/TH or 労働/AL) or (職業/TH or 職業/AL) or 人員/AL or 専門職/AL or スタッフ/AL or (職場/TH or 職場/AL) or (日常生活活動/TH or 作業/AL) or 助手/AL or 聴覚士/AL or (救急車/TH or 救急車/AL) or (介護/TH or 介護/AL) or ([臨床医]/JN or 臨床医/AL) or 沿岸警備隊/AL or (歯科医師/TH or 歯科医/AL) or 刑事/AL or 災害救助/AL or (医師/TH or 医師/AL) or (在宅介護/TH or 在宅介護/AL) or (救急医療サービス/TH or 救急医療/AL) or 救命救急/AL or (消防/TH or 消防士/AL) or 第一対応者/AL or (プライマリケア医/TH or 総合診療医/TH or 一般医/AL) or (保健医療従事者/TH or 医療従事者/AL) or (保健師/TH or 保健師/AL) or 人道支援/AL or 法執行/AL or ライフセーバー/AL or 救助員/AL or (医師臨床研修/TH or 研修医/AL) or 軍医/AL or (看護師/TH or 看護師/AL) or (助産師/TH or 助産師/AL) or (救急医療技術者/TH or 救急救命士/AL) or (警察/TH or 警察官/AL) or (薬剤師/TH or 薬剤師/AL) or (心理職/TH or 心理士/AL) or 心理師/AL or 家庭医/TH or 開業医/AL or レスキュー/AL or ([セラピスト]/JN or セラピスト/AL) or (獣医師/TH or 獣医/AL))

AND

(ランダム化比較試験/TH or ランダム割付け/TH or 無作為化/AL or ランダム化/AL)

AND

((情報機器/AL or (オンラインシステム/TH or オンライン/AL) or (インターネット/TH or インターネット/AL) or ウェブ/AL or (インターネット/TH or ウェブサイト/AL) or アプリ/AL or コンピューター/AL or デジタル/AL or (スマートフォン/TH or スマホ/AL) or (スマートフォン/TH or スマートフォン/AL) or ゲーム/AL or テレメディスン/AL or (遠隔医療/TH or 遠隔医療/AL) or エレクトロニックヘルス/AL or e-ヘルス/AL or モバイル/AL or (ウェアラブル電子機器/TH or ウェアラブル/AL) or スマートウォッチ/AL or (バイオセンシング技術/TH or バイオセンサー/AL) or (バイオセンシング技術/TH or バイオセンシング/AL) or 非接触/AL or (脳波/TH or 脳波/AL) or (脳波記録法/TH or EEG/AL) or 核磁気共鳴画像法/AL or (MRI/TH or MRI/AL) or (ニューロフィードバック/TH or ニューロフィードバック/AL) or ブレインマシン/AL or fNIRS/AL or (アルファ波/TH or α波/AL) or (アルファ波/TH or アルファ波/AL) or (不整脈/TH or 不整脈/AL) or (心拍出量/TH or 心拍出量/AL) or (脈波解析/TH or 脈波/AL) or PPG/AL or (心電図/TH or 心電計/AL) or (心電図/TH or ECG/AL) or RRインターバル/AL or RRI/AL or 心拍ゆらぎ/AL or 心拍のゆらぎ/AL or (酸素飽和度/TH or 酸素飽和度/AL) or 心拍数計測/AL or (心拍数測定/TH or 心拍数測定/AL) or (ロボット工学/TH or ロボット工学/AL) or (ロボット工学/TH or ロボティクス/AL) or ロボット療法/AL or ロボットセラピー/AL or チャットボット/AL or chatGPT/AL or バーチャル/AL or (バーチャルリアリティー/TH or バーチャルリアリティー/AL) or (バーチャルリアリティー/TH or VR/AL) or AR/AL or XR/AL or メタバース/AL or 顔認証/AL or 音声解析/AL or 音声分析/AL or (ウェーブレット解析/TH or ウェーブレット解析/AL) or (音声認識ソフトウェア/TH or 音声認識/AL) or (自然言語処理/TH or 自然言語処理/AL) or (機械学習/TH or 機械学習/AL) or (人工知能/TH or 人工知能/AL) or (深層学習/TH or 深層学習/AL) or (データマイニング/TH or データマイニング/AL) or (深層学習/TH or ディープラーニング/AL) or ("ニューラルネットワーク(コンピュータ)"/TH or ニューラルネットワーク/AL) or 信号処理/AL or 会話分析/AL or 生体信号/AL or 音声合成/AL or (モーションキャプチャー/TH or モーションキャプチャー/AL) or 三次元動作解析装置/AL or (加速度測定/TH or 加速度計/AL) or (筋電図/TH or 筋電図/AL) or (筋電図/TH or 筋電計/AL) or (筋電図/TH or EMG/AL) or モーションセンサー/AL or ジャイロ/AL or 角速度/AL or (酸素飽和度測定/TH or パルスオキシメーター/AL) or (ポリソムノグラフィー/TH or ポリソムノグラフィー/AL) or (睡眠相/TH or 睡眠ステージ/AL) or (酸素飽和度測定/TH or 酸素飽和度測定/AL) or (いびき/TH or いびき/AL) or (眼電図/TH or 眼電図/AL) or 心拍変動/AL or 睡眠効率/AL or 睡眠ポリグラフ検査/AL or (体温計/TH or 体温計/AL) or (視標追跡検査/TH or 視標追跡検査/AL) or (視標追跡検査/TH or アイトラッキング/AL) or (超音波/TH or 超音波/AL) or 光音響/AL or (光度計測法/TH or 測光/AL) or (データマイニング/TH or テキストマイニング/AL) or 機能的MRI/AL or ((@MRI/TH and @機能的神経イメージング/TH) or 機能的神経系MRI/AL) or 心拍リズム/AL or 心拍モニタリング/AL or 生活支援ロボット/AL or 顔認証/AL or (顔認識/TH or 顔認識/AL) or (顔認識/TH or 表情認識/AL) or (活動量計/TH or 活動量計/AL) or (アクチグラフィー/TH or アクチグラフィー/AL)) or ((情報機器/AL or (オンラインシステム/TH or オンライン/AL) or (インターネット/TH or インターネット/AL) or ウェブ/AL or (インターネット/TH or ウェブサイト/AL) or アプリ/AL or コンピューター/AL or デジタル/AL or (スマートフォン/TH or スマホ/AL) or (スマートフォン/TH or スマートフォン/AL) or ゲーム/AL or テレメディスン/AL or (遠隔医療/TH or 遠隔医療/AL) or エレクトロニックヘルス/AL or e-ヘルス/AL or モバイル/AL or (ウェアラブル電子機器/TH or ウェアラブル/AL) or スマートウォッチ/AL or (バイオセンシング技術/TH or バイオセンサー/AL) or (バイオセンシング技術/TH or バイオセンシング/AL) or 非接触/AL) AND ((心拍数/TH or 心拍/AL) or (呼吸/TH or 呼吸/AL) or (血圧/TH or 血圧/AL) or (身体運動/TH or エクササイズ/AL) or (体温/TH or 体温/AL) or (身体活動量の少ない生活/TH or 座位行動/AL) or (歩行運動/TH or ウォーキング/AL) or (睡眠/TH or 睡眠/AL) or (食事/TH or 食事/AL) or (栄養状態/TH or 栄養状態/AL) or 食べる/AL or エネルギー摂取/AL or (食行動/TH or 摂食行動/AL) or (CRM訓練/TH or クルー・リソース・マネジメント/AL) or (メンタリング/TH or メンタリング/AL) or 心理的安全性/AL or ストレスマネジメント/AL or チームワーク実習/AL or コミュニケーション実習/AL or コミュニケーション訓練/AL or (認知行動療法/TH or 認知行動療法/AL) or (遊戯療法/TH or 遊戯療法/AL) or (認知訓練/TH or 認知訓練/AL) or (マインドフルネス/TH or マインドフルネス/AL) or レジスタンス/AL or 座位時間/AL or (運動活性/TH or 身体活動/AL) or (食行動/TH or 食行動/AL) or (音楽療法/TH or 音楽療法/AL))))

AND

((楽観性/TH or 楽観性/AL) or (個人的満足/TH or 個人的満足/AL) or (自己概念/TH or 自己概念/AL) or (自己効力感/TH or 自己効力感/AL) or (セルフコントロール/TH or セルフコントロール/AL) or 人生への関与/AL or 人生の意味/AL or 人生の目的/AL or (楽観性/TH or ポジティブ感情/AL) or ("レジリエンス(心理学)"/TH or レジリエンス/AL) or 回復力/AL or (自己概念/TH or 自尊心/AL) or (自己概念/TH or 自尊感情/AL) or 主観的幸福感/AL or (精神的充足/TH or ウェルビーイング/AL) or (精神疾患/TH or 精神障害/AL) or (精神疾患/TH or 精神疾患/AL) or (精神保健/TH or メンタルヘルス/AL) or (産業心理学/TH or 産業心理学/AL) or (心理的ストレス/TH or 心理的ストレス/AL) or 適応/AL or (気分障害/TH or 情動障害/AL) or (気分障害/TH or 感情障害/AL) or (不安/TH or 不安/AL) or (双極性障害/TH or 躁うつ病/AL) or (双極性障害/TH or 双極性障害/AL) or (心理的燃え尽き/TH or 燃え尽き症候群/AL) or (心理的燃え尽き/TH or バーンアウト/AL) or (うつ病/TH or うつ病/AL) or (大うつ病性障害/TH or 大うつ病性障害/AL) or ((神経性やせ症/TH or 摂食障害/AL) or (摂食機能障害/TH or 摂食障害/AL)) or (神経性やせ症/TH or 拒食症/AL) or (過食/TH or 過食症/AL) or (気分障害/TH or 気分障害/AL) or (強迫症/TH or 強迫性障害/AL) or (強迫症/TH or OCD/AL) or (パニック症/TH or パニック障害/AL) or (恐怖症/TH or 恐怖症/AL) or (心的外傷/TH or 心的外傷/AL) or (ストレス障害-心的外傷後/TH or 心的外傷後ストレス障害/AL) or 精神科の診断/AL or (精神医学/TH or 精神医学/AL) or 心理的障害/AL or (心理的ストレス/TH or 心理的ストレス/AL) or (精神病/TH or 精神病/AL) or (苦痛/TH or 心理的苦痛/AL) or (ストレス障害-心的外傷後/TH or PTSD/AL) or (ストレス障害-心的外傷後/TH or PTSS/AL) or (身体症状症および関連症/TH or 身体表現性障害/AL) or (心気症/TH or 心気症/AL) or (統合失調症/TH or 統合失調症/AL) or (自傷行為/TH or 自傷行為/AL) or 自傷行動/AL or (自傷/TH or 自傷/AL) or (自殺/TH or 自殺/AL) or (物質関連障害/TH or 物質関連障害/AL) or (アルコール依存症/TH or アルコール乱用/AL) or アルコール使用/AL or (アルコール依存症/TH or アルコール依存症/AL) or (Amphetamine/TH or アンフェタミン/AL) or (大量飲酒/TH or 過剰飲酒/AL) or (アサ属/TH or 大麻/AL) or (Cocaine/TH or コカイン/AL) or (Heroin/TH or ジアセチルモルフィン/AL) or (Heroin/TH or ジアモルフィン/AL) or ((ドーピング/TH or 薬物乱用/AL) or (物質関連障害/TH or 薬物乱用/AL)) or 薬物使用/AL or (アルコール中毒-急性/TH or 酩酊/AL) or (飲酒/TH or 飲酒/AL) or (覚醒剤/TH or 覚せい剤/AL) or (恍惚/TH or エクスタシー/AL) or XTC/AL or (Fentanyl/TH or フェンタニル/AL) or ハッシシ/AL or (Heroin/TH or ヘロイン/AL) or マリフアナ/AL or (N-Methyl-3,4-methylenedioxyamphetamine/TH or MDMA/AL) or (Methadone/TH or メタドン/AL) or (Methamphetamine/TH or メタンフェタミン/AL) or (Morphine/TH or モルヒネ/AL) or ((オピオイド系鎮痛剤/TH or オピオイド/AL) or ("Opioid Peptides"/TH or オピオイド/AL)) or (アヘン/TH or アヘン/AL) or THC/AL or クリスタルメス/AL or (生活の質/TH or 生活の質/AL) or (質調整生存年/TH or 質調整生存年/AL) or (質調整生存年/TH or QALY/AL) or (障害調整生存年/TH or DALY/AL) or 機能/AL or (生活の質/TH or HRQOL/AL) or 健康関連QOL/AL or 生活活動/AL or 生活参加/AL or 生活ストレス/AL or (生活の質/TH or QOL/AL) or QOLI/AL or ((自己管理/TH or セルフケア/AL) or (自立生活/TH or セルフケア/AL) or (慢性疾患セルフマネジメント/TH or セルフケア/AL)) or 疾患影響プロファイル/AL or (社会的相互作用/TH or 社会的機能/AL) or (社会参加/TH or 社会参加/AL) or (欠勤/TH or アブセンティズム/AL) or アブセンティーズム/AL or アブセンティーイズム/AL or (欠勤/TH or 欠勤/AL) or (雇用/TH or 雇用/AL) or 職務満足感/AL or (病気休業/TH or 病気休業/AL) or (退職/TH or 退職/AL) or (作業能力評価/TH or 作業能力評価/AL) or (欠勤/TH or 欠勤/AL) or (復職/TH or 職場復帰/AL) or (復職/TH or 復職/AL) or 早期退職/AL or 雇用能力/AL or エンプロイアビリティ/AL or 雇用状況/AL or 雇用期間/AL or 雇用の維持/AL or (診断書/TH or 診断書/AL) or (作業効率/TH or 生産性/AL) or (労働時間/TH or 長時間労働/AL) or 辞職/AL or 疾病休業/AL or (欠勤/TH or 欠勤/AL) or 持続可能な労働/AL or 持続的な労働/AL or (失業/TH or 失業/AL) or 仕事の能力/AL or 職務能力/AL or (ワークエンゲージメント/TH or ワーク・エンゲイジメント/AL) or 労働機能/AL or 労働参加/AL or パフォーマンス/AL or 仕事の維持/AL or (退職/TH or 離職/AL))
