# Supplementary material for: Effects of Self-Guided Digital Mindfulness Interventions on the Mental Health of Health Care Workers: Systematic Review and Meta-Analysis of Randomized Controlled Trials
Source: JMIR Mhealth Uhealth. 2026 Aug 21;14:e74272. doi: 10.2196/74272 (PMC13494152; doi:10.2196/74272)
Supplement: Supplementary file 3 [file mhealth-v14-e74272-s003.docx]

**Supplementary Figure 1. Results of funnel plots of the included studies.**

A. Mental health symptoms & disorders

1) Depression

2) Anxiety

3) Perceived stress (Post)

3) Perceived stress (6-month Follow-up)

B. Emotional exhaustion & compassion fatigue

C. Positive mental health & well-being
