# Supplementary material for: Effects of Self-Guided Digital Mindfulness Interventions on the Mental Health of Health Care Workers: Systematic Review and Meta-Analysis of Randomized Controlled Trials
Source: JMIR Mhealth Uhealth. 2026 Aug 21;14:e74272. doi: 10.2196/74272 (PMC13494152; doi:10.2196/74272)
Supplement: Supplementary file 4 [file mhealth-v14-e74272-s004.docx]

**Supplementary File 3. GRADE Evidence profile.**

| Certainty assessment | | | | | | | № of participants | Effect | Certainty | Importance |
| --- | --- | --- | --- | --- | --- | --- | --- | --- | --- | --- |
| № of studies | Study design | Risk of bias | Inconsistency | Indirectness | Imprecision | Other considerations |  | Absolute (95% CI) |  |  |
| **Outcome: depression (meta-analysis conducted)** | | | | | | | | | | |
| 5 | RCT | Very Serious | Serious | Not serious | Not serious | None | 1,380 | SMD −0.44  (−0.88 to 0.003) | ⨁◯◯◯ Very Low | Critical |
| **Outcome: anxiety (meta-analysis conducted)** | | | | | | | | | | |
| 5 | RCT | Very Serious | Not serious | Not serious | Not serious | None | 1,380 | SMD −0.29  (−0.51 to −0.06) | ⨁⨁◯◯ Low | Critical |
| **Outcome: perceived stress (meta-analysis conducted)** | | | | | | | | | | |
| 6 | RCT | Very Serious | Serious | Not serious | Not serious | None | 1,712 | SMD −0.42  (−0.77 to −0.06) | ⨁◯◯◯ Very Low | Critical |
| **Outcome: emotional exhaustion and compassion fatigue (meta-analysis conducted)** | | | | | | | | | | |
| 4 | RCT | Very Serious | Not serious | Not serious | Serious | None | 1,535 | SMD −0.18  (−0.45 to 0.08) | ⨁◯◯◯ Very Low | Important |
| **Outcome: positive mental health and well-being (meta-analysis conducted)** | | | | | | | | | | |
| 4 | RCT | Very Serious | Not serious | Not serious | Not serious | None | 1,407 | SMD 0.20  (0.09 to 0.30) | ⨁⨁◯◯ Low | Important |

RCT=Randomized Controlled Trial.

GRADE=Grading of Recommendations Assessment, Development and Evaluation.
